# An Overview of the Function and Maintenance of Sexual Reproduction in Dikaryotic Fungi

**DOI:** 10.3389/fmicb.2018.00503

**Published:** 2018-03-21

**Authors:** R. M. Wallen, Michael H. Perlin

**Affiliations:** Department of Biology, University of Louisville, Louisville, KY, United States

**Keywords:** mating type, dimorphic fungi, *Dikarya*, bipolar mating, tetrapolar mating, pheromone/receptor, homeodomain transcription factor

## Abstract

Sexual reproduction likely evolved as protection from environmental stresses, specifically, to repair DNA damage, often via homologous recombination. In higher eukaryotes, meiosis and the production of gametes with allelic combinations different from parental type provides the side effect of increased genetic variation. In fungi it appears that while the maintenance of meiosis is paramount for success, outcrossing is not a driving force. In the subkingdom *Dikarya*, fungal members are characterized by existence of a dikaryon for extended stages within the life cycle. Such fungi possess functional or, in some cases, relictual, loci that govern sexual reproduction between members of their own species. All mating systems identified so far in the *Dikarya* employ a pheromone/receptor system for haploid organisms to recognize a compatible mating partner, although the paradigm in the Ascomycota, e.g., *Saccharomyces cerevisiae*, is that genes for the pheromone precursor and receptor are not found in the mating-type locus but rather are regulated by its products. Similarly, the mating systems in the Ascomycota are bipolar, with two non-allelic idiomorphs expressed in cells of opposite mating type. In contrast, for the Basidiomycota, both bipolar and tetrapolar mating systems have been well characterized; further, at least one locus directly encodes the pheromone precursor and the receptor for the pheromone of a different mating type, while a separate locus encodes proteins that may regulate the first locus and/or additional genes required for downstream events. Heterozygosity at both of two unlinked loci is required for cells to productively mate in tetrapolar systems, whereas in bipolar systems the two loci are tightly linked. Finally, a trade-off exists in wild fungal populations between sexual reproduction and the associated costs, with adverse conditions leading to mating. For fungal mammal pathogens, the products of sexual reproduction can be targets for the host immune system. The opposite appears true for phytopathogenic fungi, where mating and pathogenicity are inextricably linked. Here, we explore, compare, and contrast different strategies used among the *Dikarya*, both saprophytic and pathogenic fungi, and highlight differences between pathogens of mammals and pathogens of plants, providing context for selective pressures acting on this interesting group of fungi.

## Introduction

Fungi, due to their ease of manipulation in a laboratory setting and short generation time, provide excellent model organisms for studying the development of eukaryotic-specific processes, such as sexual reproduction. Unlike most higher eukaryotes, many members of this group also reproduce asexually. As such, several of these species exist as both haploid and diploid forms, whereas in higher eukaryotes such as mammals the adults are always diploid, producing haploid gametes that combine to give rise to the next generation. The maintenance of both strategies of reproduction within a single organism allows the study of both why a mechanism such as sexual reproduction may have evolved as well as why asexual reproduction may have been maintained. Ultimately, the broader question becomes can meiosis function as more than a means of production of reproductive cells.

In prokaryotic cells, conjugation and transformation serve as the means of generating recombinant genetic material in the absence of a meiotic pathway. Most of the evidence suggests that prokaryotic sex has the most value as a means of response to DNA damage (Michod et al., 2008). It is possible that meiosis emerged from transformation as a DNA repair mechanism. In lower eukaryotes, such as the algal species *Volvox cateri*, sexual reproduction has been found to be a means of responding to reactive oxygen species and the resultant DNA damage (Nedelcu and Michod, 2003). This alga, like many of the fungal species in this review, is a facultative sexual species, meaning that sexual reproduction is not an obligate component of their life cycle. Similarly, depletion of the nitrogen source in the growth medium of the unicellular green alga, *Chlamydomonas reinhardtii*, leads to differentiation of vegetative cells into gametes (Sager and Granick, 1954), which can mate, form diploids and subsequently undergo meiosis. Examination of meiosis and/or mating in the fungal subkingdom *Dikarya* also provides support for the idea that meiosis serves as a means of DNA damage repair (Bernstein and Johns, 1989; Lin et al., 2005; Steinboeck et al., 2010). However, due to the diverse lifestyles of these fungi, it is also possible to examine subsequent steps where the products of meiosis are involved in sexual reproduction. The timing of meiosis and the frequency with which in happens in each of these fungal populations is largely correlated with whether or not the organism is a pathogen.

Fungi have generally been classified into phyla based on the type of sexual reproduction a particular species uses as well as the amount of time they spend in the sexual reproductive stage. Molecular data have provided new tools for classifying fungi and introduced new complications since, depending on the subset of the molecular data chosen, the outcomes of phylogenetic classification may be different. However, it is generally accepted based on available molecular data that the *Dikarya* are composed of two monophyletic groups, the Ascomycota and the Basidiomycota (Ebersberger et al., 2012). These two groups, including industrially essential organisms such as baker’s yeast and brewer’s yeast, as well as many agriculturally important pathogens such as rusts and smuts, are potentially the most highly studied among fungi. Although the emergence of more specific taxa within each of these two groups of *Dikarya* is more difficult to discern (Ebersberger et al., 2012), examination of representative organisms within this group is useful in understanding the organisms as a whole. Particularly by examining parasites with very narrow ranges of host specificity, the interplay between the costs and benefits of meiosis within a host–pathogen system can be examined. For this reason, we will not cover mating systems of rust fungi extensively in this review; moreover, in the rust fungi, there is extensive host switching and monokaryotic stages that are pathogenic. Because of the high volume of molecular data available for these organisms as well as the differences in lifestyle amongst the two groups, *Ascomycota* and *Basidiomycota*, comparing the evolution of sexual reproduction within the two groups provides insight into the reasons this lifestyle evolved in eukaryotes.

## Genetic Components of Meiosis and Sexual Reproduction

Meiosis in fungi occurs at a different point in the reproductive lifecycle than in other higher eukaryotes. In mammals, for example, haploid gametes are produced by meiosis and then the gametes combine to form a zygote that develops into the offspring organism. Sexual reproduction in fungi occurs in three stages. First, haploid cells of compatible mating types fuse (plasmogamy). This is followed by the fusion of the two haploid nuclei (karyogamy). The newly-produced diploid cell can undergo meiosis to regenerate haploid cells, and this often is as a response to nutrient limitation (Neiman, 2011). The preliminary fusion of haploid cells requires that the cells be of compatible (usually opposite) mating type, much in the same way that two sperm or two eggs do not naturally fuse to form a zygote. What defines the mating type of a particular cell is governed by the genetic material at the mating-type locus.

In contrast to higher eukaryotes (with a few exceptions), single-celled fungi that reproduce sexually do not exist as male and female phenotypes. Rather, they exist as one of multiple mating types that are generally indistinguishable except at a molecular level. In order to have multiple mating types, there are a few essential genetic ingredients. First, an organism needs a mechanism by which to identify a member of its species of different mating type. This mechanism may be as simple as a pheromone produced by one mating type that is recognized by a member of a different mating type, or it may involve more complex mechanisms. Second, an organism needs a mechanism by which to express other genes specific to its mating type, which is usually accomplished by specialized transcription factors.

A recent review of fungal mating systems in general analyzed the complex events that have given rise to the various forms of mating type loci found in the *Dikarya*, including the existence of tetrapolar and bipolar systems (Heitman et al., 2017). The purpose of this review is to understand how the mating type loci relate to the ecological niche of each species as a means of understanding the functionality of sexual reproduction (or in some cases lack thereof) in the representative species chosen. By looking for evidence of these components within various members of the Ascomycota and Basidiomycota and considering the complexity of the mating system with respect to other aspects of life style, such as whether or not the organism is a pathogen and requires a host to complete its lifecycle, or is a free-living organism, the function of sexual reproduction for fungi can be better understood. Because these fungi may exist as either haploid or diploid organisms in natural populations, the products of meiosis are not always destined for sexual reproduction.

## The Ascomycota

The phylum *Ascomycota* or sac fungus contains a variety of organisms including plant, insect, and mammalian pathogens, as well as unicellular yeast, saprotrophs and mutualistic symbiotes. These fungi form meiotic spores called ascospores enclosed in a special sac called an ascus. As a group, sexual reproduction is more common in plant pathogens than in human pathogens and mating type (MAT) loci are comparatively small, encoding only transcription factors while pheromone and pheromone receptors are located elsewhere in the genome (Morrow and Fraser, 2009), but evidence of the genetic components for sexual reproduction exists in all members. Evidence of the genetic requirements for mating coupled with the lifestyle of the organism can provide insight into the reason for the use (or lack of use) of sexual reproduction.

### Saprotrophic Ascomycota

Saprophytic fungi are those that get their resources by degrading dead or dying organic material. Unlike pathogenic organisms, there is less evolutionary pressure supplied by the changes occurring in a host organism to which the fungi must respond. Many of the selective pressures in this group are applied by other microorganisms occupying the same environment and/or intraspecies competition for available resources. As such, the evolution and maintenance of meiosis and sexual reproduction within these fungi represents the most basic of functions and generally supports the hypothesis that sex, and more specifically meiosis, arose as a means of DNA repair following the exposure to adverse environmental conditions. However, because of the simplicity of the evolutionary pressures on these particular mechanisms, these saprophytic ascomycete fungi provide a better understanding of the role that the mating type loci play in governing meiosis and plasticity of response to changing environmental conditions.

#### Saccharomyces cerevisiae

*Saccharomyces cerevisiae*, or brewer’s yeast, is potentially the most studied eukaryotic organism. As early as the 1940s, long before molecular genetic techniques were available, scientists realized that different haploid cells of this yeast were able to mate with cells of opposite mating type (Lindegren, 1945). Despite the presumed ability to mate, *Sa. cerevisiae* is often found in nature as haploid or diploid cells that reproduce by budding. To further complicate what seems like an uncomplicated model organism, haploids may mate to form diploids, and these diploids may either undergo meiosis to form haploid spores or may themselves reproduce by budding. Not until the 1980s was the molecular mechanism of this mating more fully understood. Mating type in *Sa. cerevisiae* is determined by a single locus, the mating type locus defined as either *MATa* or *MAT*α, based on the resident sequence. However, it was discovered that haploid cells of one mating type (either **a** or α) could produce progeny of the opposite mating type during mitosis (Hicks et al., 1979). These findings led the development of the “cassette model” of mating type switching, in which “silent” copies of the alternate mating type resided in another location within the genome (Hicks et al., 1979). Haploid cells of mating type **a**, for example, were found to contain all the genetic elements to be mating type α, but the genetic material was contained in a transcriptionally non-active region within the genome (Hicks et al., 1979).

The genetic regions at either *MAT****a*** or *MAT*α were later characterized and the function of the resulting proteins analyzed. The two regions are not the same length, with *MAT****a*** being shorter than *MAT*α; they encode regulatory proteins that control α-specific or **a**-specific genes (Astell et al., 1981). The two versions share little homology and so, are not true alleles, but rather are termed “idiomorphs” (Metzenberg and Glass, 1990). In fact, all of the proteins encoded by the *MAT* locus are DNA binding proteins which define one of three cell types (haploid *MAT*α or *MAT****a***, or diploid) by directing other gene expression (Dranginis, 1990). The activity of products of the *MAT*α locus determines whether the cell exhibits α or **a** phenotype. *MAT*α encodes two genes, *α1* and *α2*. MATα1 controls expression of α-specific genes, while MATα2 suppresses **a**-specific genes that would otherwise be expressed constitutively (Astell et al., 1981; Dranginis, 1990). MATα2 binds as a dimer to DNA with the assistance of other factors in haploid α2 cells to block expression of **a**-specific genes, including **a**-specific pheromones and receptors described below (Zhong and Vershon, 1997). The *MAT****a*** locus encodes a single protein, a1, which is dispensable for mating, but acts with α2 to control gene expression after mating has occurred in the diploid state (Astell et al., 1981). In the diploid, α2 and a1 form a DNA-binding heterodimer. The α2 protein contains a homeodomain region plus an additional sequence at the C terminus while the a1 contains only the homeodomain motif, suggesting that a1 mediates both DNA binding and interaction with α2 (Phillips et al., 1994). In a/α diploid cells, the heterodimer represses expression of haploid specific genes (Dranginis, 1990).

In addition to the genetic requirement to have a set of genes specific to mating type, *Sa. cerevisiae* also has a system by which to identify cells of the opposite mating type in the form of a pheromone/pheromone receptor system. While in this organism, as is typical with other Ascomycota, the pheromone/pheromone receptor system is not part of the mating type locus, products of the mating type locus can affect expression of the pheromone and receptor genes. Cells that exhibit the **a** phenotype express a-factor (either Mfa1 or Mfa2), a polypeptide hormone or pheromone; the encoded precursor is processed via several proteolytic cleavages and is modified via prenylation and carboxymethylation prior to export (Michaelis and Barrowman, 2012). The exported **a**-factor interacts with α cells to cause cell cycle arrest and other physiological changes associated with the mating response (Michaelis and Herskowitz, 1988). In α haploid cells, α-factor, a pheromone, is produced which also interacts with **a** haploid cells to arrest the cell cycle prior to DNA synthesis (Bücking-Throm et al., 1973). This arrest in cell cycle, specifically G1, synchronizes haploid cells in preparation for conjugation (Wilkinson and Pringle, 1974). Haploid cells also produce receptors that recognize the pheromones from haploids of opposite mating type. In α haploid cells, *ste3* encodes the receptor for a-factor. It is required for mating only in α cells and its transcription only occurs in α cells (Hagen et al., 1986). Moreover, **a**-factor is able to induce the production of its receptor in α cells (Hagen and Sprague, 1984). In **a** haploid cells, *ste2* encodes the receptor for α-factor.

**FIGURE 1:**
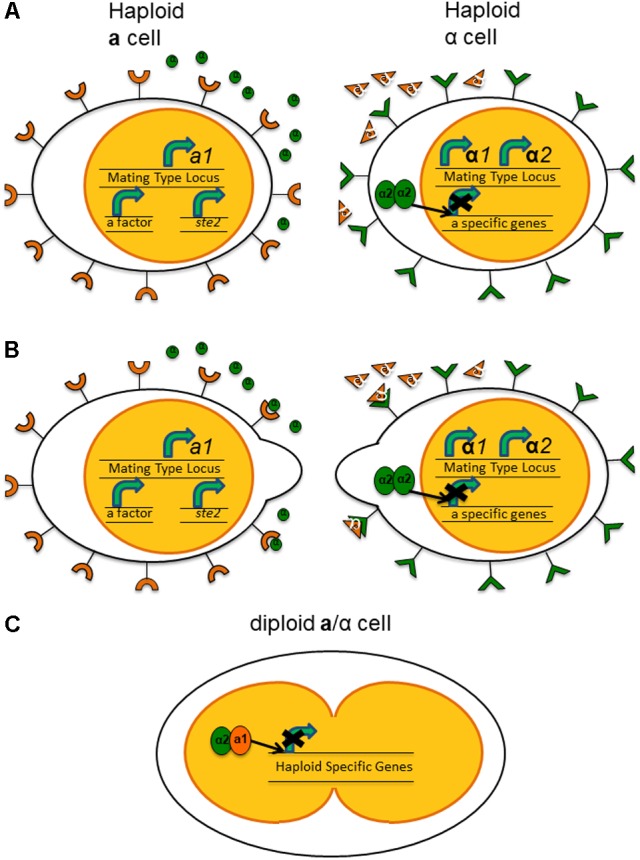
Expression of haploid-specific genes in *Saccharomyces cerevisiae* leading to mating between haploid cells. **(A)** In the haploid **a** cells, *a1*, the homeodomain protein, is expressed constitutively from the mating type locus. While the genes for the pheromone and pheromone receptor are not located within the mating-type loci, in the haploid **a** cell, the a-factor and Ste2, the receptor for α-factor, are both expressed constitutively as well. In the haploid α cells, *α1* and *α2* are expressed from the mating type locus. α2 forms a homodimer to block transcription of **a**-specific genes. **(B)** The interaction of pheromone with its receptor on the cell of the opposite mating type results in the formation of a “shmoo” structure, a protrusion of the cytoplasm, toward each other. **(C)** Fusion of the cells and subsequently the nuclei. In the diploid a/α cell, α2 and a1 form a heterodimer to block expression of haploid-specific genes.

Exposure to the pheromone of the opposite mating type not only arrests the cell cycle but also changes expression of a variety of genes. For example, when exposed to α-factor, *MATa* haploids show decreased expression of genes related to vegetative growth and increased expression of genes related to the mating process (Stetler and Thorner, 1984). Additionally, while the pheromone/pheromone receptor system is not physically part of the mating type loci in *Sa. cerevisiae*, the activity of the products of the *MAT* can affect their expression level. MATα1 activates transcription of α-specific structural genes, including **a**-factor receptor genes, while MATα2 represses a-specific structural genes and α-factor receptor genes (Bender and Sprague, 1989). Binding of the pheromone to its appropriate receptor sets off a signaling cascade that leads to changes in global gene expression and physiological changes in the haploid cells (van Drogen et al., 2001). The haploid cells change the polarity of growth, growing toward each other, forming a “shmoo” or bulging extension of their cell wall and cytoplasm in the direction of the cell of opposite mating type. These formations will eventually join, forming a conjugation tube and leading to the fusing of both the cells and their nuclei in the diploid cell (**Figure 1**). Meiosis of this diploid then produces ascospores, each containing a haploid nucleus. The spores are more resilient to adverse environmental conditions than the vegetative yeast (Taxis et al., 2005).

The presence of the components of a mating system, in this case, do not dictate a single strategy by which this particular yeast is able to reproduce. *Sa. cerevisiae* can reproduce asexually from either the diploid or haploid form. Haploid spores produced from meiosis from a diploid strain can mate within the ascus, and haploid spores from genetically divergent strains could also mate should they encounter one another (Ruderfer et al., 2006). Despite the presence of all the genetic requirements necessary for a sexual reproduction system, outcrossing (that is, mating with a genetically divergent mating partner) in *Sa. cerevisiae* in wild populations is relatively rare. Genetic analysis of several wild isolates estimates that this yeast mates approximately once every 50,000 divisions (Ruderfer et al., 2006). The same study estimated that any two strains have undergone approximately 16 million cell divisions since their last common ancestor with only 314 outcrossing events (Ruderfer et al., 2006).

Because *Sa. cerevisiae* generally are saprophytic and require no interaction with a live host, the maintenance of a mating system is not driven by the need to create variability in the population to evolve along with the host’s ability to rid itself of the invading pathogen. However, the yeast nonetheless requires the ability to respond to changes in its environment and mating may provide the means by which to do so. Diploid *Sa. cerevisiae* cells, resulting from mating, reproduce mitotically when nutrient availability is high, but undergo meiosis when starved. Such starvation increases oxidative stress and DNA damage, in the form of double-strand breaks and accumulation of sites lacking bases (Steinboeck et al., 2010). Could induction of sex by starvation be mediated by oxidative stress, similar to what was seen for heat shock in *V. cateri*, discussed above? That effect can be inhibited by antioxidants, indicating that induction of sex by heat shock is mediated by oxidative stress (Nedelcu and Michod, 2003). The *Sa. cerevisiae* Rad52 protein promotes the DNA strand exchange reaction of recombination during meiosis and mitosis (Mortensen et al., 2009). *Sa. cerevisiae rad52* mutants are sensitive to killing by several DNA damaging agents. Diploid cells of *Sa. cerevisiae* are able to repair DNA double strand breaks due to ionizing radiation, and this ability is lost in mutant strains defective in the *rad52* gene (Resnick and Martin, 1976). *Sa. cerevisiae* can also undergo changes in cell shape and patterns of cell division leading to formation of long, thin pseudohyphae that grow away from the central colony (Gimeno et al., 1992). This phenomenon is only observed in diploid cells and does not happen with haploid cells (Gimeno et al., 1992). In the lab setting on replete media, *Sa. cerevisiae* will mate spontaneously when grown with members of the opposite mating type, forming stable diploids (Merlini et al., 2013). In starvation conditions such as low ammonium and poor carbon sources, diploid cells undergo meiosis to form spores (Neiman, 2011; Merlini et al., 2013). The products of meiosis are also affected by environmental conditions, as the number of ascospores produced during sporulation is decreased in carbon-limiting conditions (Taxis et al., 2005).

Since *Sa. cerevisiae* has the ability to change mating type, this model system also illustrates the importance of examining wild populations of fungal species, not just the behavior of such organisms in the lab, to better understand the lifestyle subjected to evolutionary pressures. Due to the ability to switch mating type, this system may on its surface appear to be homothallic, as both mating types could be produced from a single cell. However, examination of wild populations where mating-type switching is theoretically possible, shows that mating-type in relatively stable and heterothallism the preferred strategy (Ezov et al., 2009). This further illustrates the importance of meiosis to this fungal system. In a homothallic system, compatible mating partners could be created through a mitotic event (e.g., mother–daughter mating). Heterothallism, however, requires meiosis, although mating may occur between members of the same meiotic tetrad.

Further evidence of the use of meiosis as a means of DNA damage repair can be found in the activity of certain DNA repair mechanisms in *Sa. cerevisiae* only during meiosis. For example, *rad2* transcript levels are elevated during meiosis and remain relatively constant during mitosis. The product of this gene, along with several other closely related proteins, function in the DNA excision repair mechanism and may be pivotal to the completion of recombination (Madura and Prakash, 1990). Moreover, five out of the six homologs for prokaryotic MutS, part of the DNA mismatch repair system, have been found to function during meiosis in *Sa. cerevisiae* and mutants defective in any of these five show marked inability to repair mismatched DNA pairings (Sia and Kirkpatrick, 2005).

As a generalization, we can say that the main function of sexual reproduction in all organisms is DNA repair and production of higher quality offspring (Horandl, 2009). In this fungal system, the occurrence of meiosis allows for DNA damage repair in the form of cross-over or other forms of recombination; however, the products of meiosis are not necessarily destined for outcrossing. Nutrient starvation, such as nitrogen starvation, can induce damage to an organism’s DNA. Mitophagy, or the autophagy-dependent destruction of mitochondria, is initiated when wild-type yeast are starved for nitrogen, resulting in the production of reactive oxygen species, known to cause DNA damage (Kurihara et al., 2012). Haploid cells are not capable of undergoing meiosis to address the potential damage done to DNA by nutrient limiting conditions. Only diploid cells, the product of two mated haploids, are capable of such a process. In the system, the components of the mating type loci both govern the ability to mate and ultimately the genetic material to be repaired through meiosis and the phenotypic changes that take place allowing the organism to “scavenge” nutrients in its environment by increasing its surface area through the growth of pseudohyphal appendages. Meiosis, only possible by the combination of different mating type loci, allows for DNA damage repair but not necessarily an increase in genetic diversity. Because the mate does not have to be from a genetically divergent strain, but could be a member of the meiotic tetrad, finding a mate is not a limiting factor in responding to nutrient starvation. Rather, the maintenance of sexual reproduction in the absence of outcrossing ensures the survival of the species and dynamic response to changing environmental conditions.

#### Schizosaccharomyces pombe

Another ascomycete fungus provides additional evidence for the concept of sexual reproduction evolving as a mechanism of response to low nutrient availability. *Schizosaccharomyces pombe*, or fission yeast, is also a saprophytic yeast, requiring dead or decaying organic matter, but not a live host, and exists in two mating types h+ and h-. *Sc. pombe*, like *Sa. cerevisiae*, has a relatively simple life cycle consisting of a haploid and diploid state (Kelly et al., 1988). However, unlike *Sa. cerevisiae*, the diploid form of *Sc. pombe* is unstable, although it can be maintained in the lab on nitrogen replete media (Wells et al., 2006).

The two requisite groups of genetic elements, transcription factors to express mating-type specific genes and a pheromone receptor system are also present. The mating type region of *Sc. pombe* consists of three components, *mat1*, *mat2-P* and *mat3-M* (Kelly et al., 1988). Cell type is determined by alternate alleles present at the *mat1* locus, either P in h+ cells or M in h- cells. The P region contains two open reading frames Pc and Pi. The predicted product of Pi contains regions homologous to homeobox sequences and is therefore believed to encode DNA binding proteins that regulate the expression of other genes (Kelly et al., 1988). The M region also contains two open reading frames Mc and Mi, and Mc also appears to have homeobox like sequences (Sugimoto et al., 1990). As with *Sa. cerevisiae*, not all products of the mating type locus are necessary for both haploid and diploid cell types. Pc and Mc alone were necessary to produce h+ and h- phenotypes, respectively, in haploid cells but all four, Pc, Pi, Mc, and Mi were required for gene expression in the diploid.

Like *Sa. cerevisiae*, haploid *Sc. pombe* cells secrete pheromones that are recognized by cells of the opposite mating type. Haploid h+ cells secrete P factor and h- cells respond to this pheromone in a physiological manner (Imai and Yamamoto, 1994). Similarly, haploid h- cells secrete M factor to which h+ cells respond (Davey, 1991). Like budding yeast, fission yeast also respond to pheromone stimulation with cell cycle arrest. Interestingly, in this system, it was originally believed to be nutrient starvation that arrested cell cycle, not response to pheromones (Imai and Yamamoto, 1994). In addition to pheromones stimulating cell cycle arrest and fusion of two haploid cells, in this system pheromone stimulation has also found to be required for appropriate arrangement of chromosomes during meiosis to allow for recombination (Wells et al., 2006).

While nutrient starvation is not directly responsible for cell cycle arrest, the responsiveness of mating-related genes to nutrient starvation provides additional evidence for the evolution and maintenance of mating system as a response to nutrient starvation. While Pc and Mc are expressed from the mating type locus at a low basal level, expression of both is greatly induced in nitrogen-free media and expression of Pi and Mi is only detectable under conditions of nitrogen starvation (Kelly et al., 1988). Furthermore, nutritional starvation also leads to decreased levels of cAMP, an essential signaling component in mating pathways (Mochizuki and Yamamoto, 1992). Although nitrogen starvation seemed to induce transcription from mating type alleles, the response to starvation of all nutrients was not the same. Glucose-depleted but nitrogen-replete media did not result in the same induction of *mat* transcripts (Kelly et al., 1988). Additionally, another protein, Ste11, is induced in response to nitrogen starvation and decreased cAMP levels (Sugimoto et al., 1990). Like Ste11 in *Sa. cerevisiae*, the *Sc. pombe* protein is essential for sexual development. Ectopic exposure to Ste11 causes uncontrolled mating and sporulation in *Sc. pombe*. Unlike the protein of the same name in *Sa. cerevisiae*, which is a protein kinase, *Sc. pombe* Ste11 encodes an HMG-box protein and regulates transcription of *matP* and *matM*. While not part of the mating type loci, this *Sc. pombe* protein represents another tie between nitrogen starvation and meiosis. The regulation of mating-type related genes by HMG-box proteins outside of the mating-type locus also appears in other ascomycete fungi and is pivotal to sexual development (Ait Benkhali et al., 2013).

Further evidence for meiosis as a means of DNA repair also comes from responses of *Sc. pombe* to several forms of stress. The fungus is induced to undergo sexual development and mating when the supply of nutrients becomes limiting (Davey, 1991). Moreover, oxidative stress induced in late exponential vegetative cells by exposure to hydrogen peroxide, increases the frequency of mating and production of meiotic spores (Bernstein and Johns, 1989), a response that would be expected if mating and subsequent meiosis have evolved as a DNA repair mechanism in response to stress (Bernstein and Johns, 1989).

#### Neurospora crassa

**FIGURE 2:**
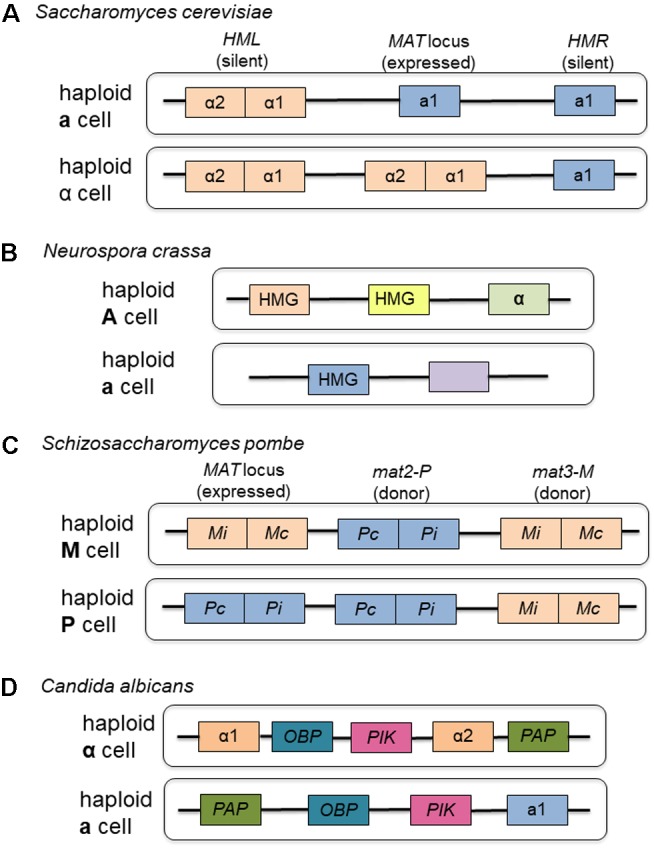
Representative mating type loci from the Ascomycota. **(A)** The organization of the mating-type loci of *Saccharomyces cerevisiae* serves as the model for the group, containing homeodomain proteins that regulate the expression of other mating type-specific genes. **(B)** Although not as well characterized as *Sa. cerevisiae*, *Neurospora crassa* exists as two haploid mating types, each of which contain genetic information at the mating type loci to govern expression of mating type specific genes. **(C)** Like *Sa. cerevisiae*, *Schizosaccharomyces pombe* contains homeodomain proteins at its mating type loci consisting of both expressed and donor regions. **(D)** Although most clinical isolates are diploid, *Candida albicans* does have two different mating type loci found in two haploid strains that can be cultivated in the laboratory setting. Boxes of the same color represent corresponding components in the different mating types.

As the model organism for the Ascomycota, the mating type locus of *Sa. cerevisiae* is the standard by which all other mating type loci of the group are measured. While other sac fungi use components of their mating type loci to a greater or lesser extent, the components are highly conserved across the group (see **Figure 2** for a comparison of representative Ascomycete mating type loci). A saprophytic bread mold, *Neurospora crassa*, also provides evidence for the close relationship between nutrient availability and the ability to undergo meiosis following mating. Before the elements of the mating type locus and developmental program of this ascomycete fungus were fully understood, researchers recognized that in order to induce mating, this organism required low nitrogen, low light and low temperature (Perkins and Berry, 1977), all conditions unfavorable to vegetative growth. Subsequent research demonstrated that the physiological changes necessary for mating, such as production of the female mating structures, protoperithecia and trichogynes, and vegetative spores or conidia, also happened under depleted environmental conditions (Staben and Yanofsky, 1990). The fusion of the trichogynes to conidia of opposite mating type initiates sexual reproduction and maturation of sexual structures, and these processes are governed by components of the mating type locus (Staben and Yanofsky, 1990). Trichogynes adjust their growth pattern in the direction of conidia of opposite mating type, also governed by products of the mating type locus (Bistis, 1981).

Like other ascomycete fungi, the mating type locus contains genetic elements that allow for recognition of a cell of the opposite mating type and for expression of mating type specific genes. They also control entry into the sexual cycle and prevent formation of mixed heterokaryons during the vegetative phase (Glass et al., 1990). The mating types in *N. crassa* are termed A and a. The *A* mating type locus contains sequences responsible for recognizing cells of opposite mating type and a region encoding a high degree of amino acid similarity to MATα1 of *Sa. cerevisiae* (Glass et al., 1990). The *a* region contains at least two functional segments, one of which is responsible for maturation of sexual structures and the other of which specifies mating identity and induction of mating (Staben and Yanofsky, 1990).

Like *Sa. cerevisiae*, *N. crassa* has pheromones and pheromone receptors that function in mating but are not part of the mating type locus. The putative pheromone precursor, Mfa-1 (mating factor a-1) shows many structural similarities to other fungal pheromone precursors and is found in greatest abundance when cells are starved for nutrients (Kim et al., 2002). Unlike pheromone precursors in *Sa. cerevisiae*, products of this gene may function in both mating types, attracting trichogynes of mating type A but also functioning in the development of female structures in both mating types as well as in vegetative growth (Kim et al., 2002). The mating type A pheromone precursor, *ccg-4*, has also been shown to accumulate under low nitrogen conditions (Bobrowicz et al., 2002). Pheromone receptors have also been identified with sequence similarity to those in *Sa. cerevisiae* and mating type specific expression (Kim and Borkovich, 2004). *N. crassa* provides additional evidence for the link between starvation for nutrients, specifically nitrogen, and the induction of mating in the Ascomycota.

Unlike the yeasts, *N. crassa* exists longer in the heterokaryotic phase before nuclear fusion (Selker, 1990). Several duplications of the haploid nuclei within the heterokaryote occur before karyogamy, and once karyogamy finally does happen the resulting diploid immediately enters meiosis (Selker, 1990). In this organism, meiosis is limited to a single cell: four haploid ascospores are produced which then undergo mitosis to produce a total of eight ascospores (Selker, 1990). Analysis of its complete genome reveals that *N. crassa* also possesses a wide array of other genomic defense mechanisms, unique among eukaryotes, to prevent the accumulation of repeat sequences (Galagan et al., 2003). While the importance of DNA repair during meiosis remains a driving force for its maintenance, the cost of accumulation of unnecessary genomic elements has also resulted in the continuation of mechanisms that eliminate the possibility of an inefficient genome. For this saprophyte, quick growth not hindered by a cumbersome genome has been a strong evolutionary force driving the shape of its genomic evolution.

Evidence from these three saprophytic organisms as a group implicates the importance of meiosis following exposure to environmental conditions leading to DNA damage. As the only cells within the lifecycle of these organisms are the haploid products of meiosis, the components of the mating type loci are paramount in allowing DNA repair and are intrinsically linked to response to starvation. While these saprophytes have been selected to maintain such a process despite the lack of maintenance of a diploid state or tendency toward outcrossing, examination of pathogenic organisms reveals that other selective pressures of different ecological niches has shaped the timing of meiosis within the organism’s lifecycle and its time of interacting with its host.

### Pathogenic Ascomycota

Organisms with parasitic lifestyles undergo selective pressures from a variety of biotic and abiotic forces inside and outside of their hosts. In the case of fungal pathogens, whether of plant or animal, the organism must itself evolve to keep up with the dynamic immune response the host has to its presence while continuing to maintain its own structural and genetic integrity. Because there are a variety of ascomycete fungi that are human pathogens, it is likely that the ability to be pathogenic arose multiple times within the lineage (Bowman et al., 1992). Not all of these organisms are capable of sexual reproduction, and even those that appear to have the genetic components have been elusive in revealing the details of their meiotic cycles. However, due to the addition of evolutionary pressures applied by the host organism, the function of meiosis within the lifecycle of the organism becomes more complex.

#### Magnaporthe grisea

The *Magnaporthe grisea* and *Magnaporthe oryzae* species complex are the causative agents of rice blast disease and have a huge economic impact on the agricultural industry. Like other members of the Ascomycota, a region within the genome have been identified as the mating type locus, distinguishing the two mating types, Mat1-1 or Mat1-2. Similar to what has been found in other fungi, these regions are idiomorphs with relatively no sequence similarity (Kang et al., 1994). Additionally, pheromone precursors have been identified within the genome. While genes for both pheromone precursors are present in the genomes of both mating types, they are expressed in a mating-type specific manner. *MF1-1* is expressed by Mat1-1 strains while *MF1-2* is expressed by Mat1-2 strains (Shen et al., 1999). What is more interesting still, these pheromones are not expressed under low nutrient availability as is seen in other ascomycete fungi but under relatively replete media conditions (Shen et al., 1999).

The infectious process of the *Magnaporthe* complex may offer some insight into this deviation from normal induction of pheromone expression level. Rice blast infection begins when a haploid spore forms an appressorium and is able to penetrate plant tissue, develop, and establish infection inside the host. While many of the pathways governing this development and infection are canonical signaling pathways involved in mating in other fungi, such as cAMP/PKA pathway and MAPK pathways, there appears to be no requisite for mating in order for this organism to form an appressorium and enter the host (Choi and Dean, 1997; Zhao et al., 2005). While strains of opposite mating type can mate in a laboratory setting, most field isolates from rice are relatively infertile, although isolates from different hosts demonstrate different degrees of fertility (Li et al., 2016). Moreover, appressorium formation seems to be repressed by the presence of mating pheromones. In response to *Sa. cerevisiae* α-factor, *Mag. grisea* Mat1-2 does not form appressoria even in typically inducing environmental conditions and this lack of morphological response appears to be due to the interaction of the pheromone with the pheromone receptor. Although it was not demonstrated to specifically be the Mat1-1 pheromone, culture filtrates from Mat1-1 had the same effect on Mat1-2 appressorium formation (Beckerman et al., 1997).

Analysis of the genomes of several field isolates of *Magnaporthe* species from different hosts reveals that divergences in the fungal genome have correlated with divergences in the host plants (Zhong et al., 2016). Unlike some of the other phytopathogens to be discussed later, mating in this fungus is not a requirement for infection of the host and the infectious structure arises from a haploid cell. Moreover, this complex of fungal species has such great host specificity that an isolate from one host fails to establish infection in a different host species (Zhong et al., 2016). As such, meiosis and the potential shuffling of alleles could have catastrophic consequences to the pathogen, resulting in loss of host-specific virulence factors that would result in decreased ability to establish infection. Although the genetic components at the mating type loci have been maintained in this organism, indicating the likely ancestral state of this group, the normal mating-inducing influence of exposure to pheromones of the opposite mating type has changed to an infection supression function.

*Magnaporthe grisea* represents an extreme example of change in sexual reproduction strategies as a result of host interactions. Mating is a costly process and potentially may reduce the ability of the organism to infect its host plant. This fungus has been able to circumvent the need for mating altogether, while maintaining the pathways involved in other mating systems. Despite the evolution of a mating system and meiosis, potentially in response to DNA damage, successful infection without mating has been maintained in the population. This may be as the selective pressure in favor of infection outweighs that in favor of whatever positives can be gained from mating. While *Magnaporthe* species represent an extreme strategy of sexual reproduction (or lack thereof) to produce successful infection, other plant pathogen members of *Ascomycota* that also reproduce primarily asexually have evolved other means of using components of the mating pathway for interaction with their plant hosts.

#### Fusarium oxysporum

*Fusarium oxysporum* represents a fungal complex causing vascular wilt disease in over 100 different plant species. Because the fungus dwells in the soil and enters the plant through the root, there is some evidence indicating that the fungus can sense the host plant and grow in that direction (Turrà et al., 2015). Despite its asexual lifestyle, *F. oxysporum* still has the mating type locus components found in all the Ascomycota. There are two idiomorphs present at the mating type locus, either *MAT1-1* or *MAT1-2*. Although their function in an asexual fungal system is largely unknown, the *MAT1-2* idiomorph does encode a protein with an HMG-box domain, like other ascomycete fungi (Arie et al., 2000). When expressed in other closely related species known to have a sexual component to their life cycle, these genetic elements still allowed for mating (Arie et al., 2000). While the genetic material has not yet been removed from the genome, in this particular fungal pathogen it has ceased to function in a sexual reproduction program. Furthermore, *F. oxysporum* has been found to contain a novel type of peptide precursor found in a handful of other ascomycete fungi. While the function of this particular type of pheromone has not been fully determined, it may indicate a different type of reproductive strategy or signaling strategy in this type of asexually producing fungus (Schmoll et al., 2010).

What is particularly interesting with the species complex, in the context of understanding the evolution of *MAT* loci, is the possibility of the pheromone receptor system, and potentially the novel pheromone precursor, in sensing and/or sending chemical signals to and from potential host plants. Growth of the fungal hyphae toward the host plant is directly in response to a specific type of peroxidase secreted by the plant (Turrà et al., 2015). Moreover, exposing microconidia to glutamate results in increased production of germ tubes (Turrà et al., 2015), indicating that nitrogenous nutrients can elicit a chemotrophic response as well. This response was specific to only some amino acids and nitrogen sources, indicating that it was not a general response to any nitrogen. While no sexual cycle as been found in *F. oxysporum*, putative pheromone precursors are found in the genome, and exposure to either these pheromones or pheromones from *Sa. cerevisiae* (specifically α pheromone) resulted in a similar chemotrophic response (Turrà et al., 2015). With respect to canonical mating pathway components, this chemotrophic response was found to be mediated by a MAPK cascade as well as the *F. oxysporum* Ste2, a functional homolog of the protein of the same name is *Sa. cerevisiae* that is one of the pheromone receptors (Turrà et al., 2015). While exposure to pheromone can cause morphological changes in a responding cell, in a similar fashion, signals received by the pheromone receptor can change the growth of the fungal hyphae in the direction of a potential host. Although the pheromone receptor may no longer have a role in sexual development, it is vital to the fungal ability to sense a suitable host in its environment.

#### Candida albicans

As the most prevalent fungal pathogen of humans, *Candida albicans* provides an example of an ascomycete with the requirement for a host. Interestingly, it was long believed that this organism did not undergo any type of sexual cycle. Evidence of the genetic components for a sexual reproduction system prompted researchers to investigate further. Not until the late 1990s was it determined that *Ca. albicans* did in fact contain regions with similarity to mating type loci in *Sa. cerevisiae.* These regions, named *MTLa* and *MTL*α, both encode regulatory sequences similar to those found in the corresponding regions of *Sa. cerevisiae*, although spanning a larger area within the genome (Hull and Johnson, 1999). Within these regions several similarities were found to the *MAT* loci of other fungi, including the presence of transcriptional regulators, the general organization of the loci, and conserved position of introns within various coding regions (Hull and Johnson, 1999). In addition to the presence of transcriptional regulators, pheromone and pheromone receptor genes were also discovered within the genome (Bennett et al., 2003). In fact, response to pheromone of the opposite mating type induces the expression of many genes whose homologs in *Sa. cerevisiae* are known to be involved in mating (Bennett et al., 2003). So with all the requisite components, why was there no evidence of meiosis and mating in these fungi?

The answer seems to lie within the specific relationship this pathogen has with its host. In a lab setting, *Ca. albicans* cells can grow as white or opaque colonies, regardless of mating type. Interestingly, the switch from white to opaque is controlled by homeodomain proteins within the mating type locus (Miller and Johnson, 2002). The switch from white to opaque is relatively rare. Infrequent mating observed in clinical populations seems to be a result of the relationship between virulence and mating efficiency; that is, the type of cells that establish the most robust systemic infection are those least suited to mate (Hull and Johnson, 1999).

Generally speaking, *Ca. albicans* exists as a diploid, but genes from the mating type loci exist in only a single copy (Hull and Johnson, 1999). However, the most commonly studied lab strain exists as a heterozygote at the mating type locus, possessing both mating type alleles. Strains were genetically manipulated to create single mating-type diploids to allow the study of the response of the two cell types to each other (Miller and Johnson, 2002). Like *Sa. cerevisiae*, these mating types were termed **a** and α. These strains both produced superficially opaque colonies, although the parental diploid strain did not (Miller and Johnson, 2002). Opaque cells of α type secreted pheromones that cause opaque cells of **a** type to form projections toward the α type cells. This phenomenon was only observed in opaque cells of **a** type and not α type indicating the response was mating type-specific (Miller and Johnson, 2002; Bennett et al., 2003). Opaque cells are 10^6^ times more efficient at mating than white cells, while white cells are generally the phenotype found in mammalian infections (Miller and Johnson, 2002).

While opaque cells have much higher efficiency at mating, they are also more unstable in the host environment, which has long been used as support for the idea that *Ca. albicans* almost never mates within the human host (Slutsky et al., 1987; Rikkerink et al., 1988). Division of the human host into a variety of niches rather than an organismal approach has revealed that this assumption may only be true in certain regions of the body and may be influenced by the presence of other microbes. In the gastrointestinal tract for example, co-habitation with bacterial inhabitants and the environment that this creates could potentially induce white-to-opaque phenotype and presumably the ability to mate within the host (Huang et al., 2010). In other systems, the presence of bacteria and molecules that they secrete have been found to induce sexual reproduction in eukaryotic organisms within close proximity (Woznica et al., 2017). Although no mating within the host in this sub-niche has been observed, the theoretical possibility for mating of *Ca. albicans* within the host exists, potentially in response to the presence of other microbes.

Recent clinical isolates of *Ca. albicans* have provided evidence of a potential third phenotype, a “gray” colony formation in addition to white and opaque (Tao et al., 2014). While this phenomenon is not observed in all clinical isolates, in those that do have the ability to switch between three instead of two phenotypes, the switch is independent of regulation by the mating type locus (Tao et al., 2014). The mating competency of strains with the triphenotype variation is intermediate to those of the opaque and white phenotypes (Tao et al., 2014). As this new “gray” phenotype is not pervasive amongst all clinical isolates of *Ca. albicans*, data are not available regarding how frequently these particular strains mate either inside or outside of the host. Authors hypothesize that this additional phenotype may be the result of establishing specific niches within the human host, evolving after the suppression of switching to highly antigenic phenotypes (Tao et al., 2014).

The challenge for a human pathogen then, is to withstand the onslaught of the host immune system and compete with a variety of other microbes living within the same host. While a human pathogen must be able to survive inside its host and respond dynamically to the host’s immune system, ascospores are highly antigenic so sexual reproduction inside the host may inadvertently lead to a greater and more effective immune response (Forche et al., 2008). *Ca. albicans* has, as a result, evolved what has been termed a parasexual life cycle. This form of reproduction allows for some of the DNA repair created by recombination and none of the undesirable host attention attendant with switching to opaque cell type or producing ascospores. In this system, tetraploid strains become unstable and start losing chromosomes to generate diploid strains (Forche et al., 2008). Genetic recombination happens at a lower rate than in a normal meiotic cycle but sufficient to generate some variation within the population (Forche et al., 2008). While imprecise, parasexual reproduction does help to increase diversity and explains the maintenance of the mating-related genes within *Ca. albicans* despite the lack of obvious meiosis. Although mating within the host may be theoretically possible, it has not been demonstrated as happening frequently, regardless of the specific region of the body inhabited by the fungal pathogen.

As a mammalian pathogen, *Ca. albicans* represents an extreme example of evolution of sexual reproduction as a response to host specificity. Because the host immune response is more intense toward the phenotype of cells required for mating and for the products of mating, selection has been in favor of a parasexual reproductive life cycle that avoids both. Other human pathogens presumed to be asexual for similar reasons have actually found to still possess the ability to reproduce sexually although the occurrence in nature or within the host is not fully determined.

#### Aspergillus fumigatus

*Aspergillus fumigatus* can live saprotrophicly but can also be a human pathogen particularly in immunocompromised individuals. Because of its clinical importance, it has been the subject of much study. *A. fumigatus*, first described more than 150 years ago, is ubiquitous in soil and other types of organic debris and infection within humans has a high mortality rate (Mullins et al., 1976; Latge, 1999). Although *A. fumigatus* was believed to only reproduce as mitotic spores, mating type loci have been found in the genome, with HMG-domain genes similar to those found in other sexually reproducing species as well as pheromone and pheromone precursors (Galagan et al., 2005; Paoletti et al., 2005). Two versions of the mating type loci, designated *MAT1-1* and *MAT1-2*, have been identified, also indicating the possibility of sexual reproduction (Galagan et al., 2005; Paoletti et al., 2005). However, study of clinical and environmental isolates have uncovered an approximately equal ratio of the two mating types present, indicative of lack of sexual reproduction (O’Gorman et al., 2009).

*In vitro* experiments in the laboratory with strains of opposite mating type have indicated that this fungus is able to mate with a partner of the opposite mating type (O’Gorman et al., 2009). After identification of mating type loci and the possibility of mating, the virulence associated with particular genetic information at the mating type loci was investigated. No difference was found in either mating type as related to virulence, indicating the mating type locus plays no as yet discovered role in ability to infect (Losada et al., 2015). This finding is consistent with a lack of one mating type or the other being more prevalent in clinical isolates.

Manipulation of this fungal opportunistic pathogen has uncovered a variety of useful molecular techniques for understanding the sexual life cycle of organisms that appear to have none. It is important to note that mating in the controlled setting of the lab does not necessarily translate to mating in the host environment. The ability of *A. fumigatus* to mate within the human host has not yet been discovered and as such, the importance of mating in virulence remains elusive. Like *Ca. albicans*, mating may be specific to certain physiological conditions found within the human host, or may be lost all together as part of the modern lifecycle given the success the organism has in reproducing by mitosis alone.

As with some plant pathogens within the Ascomycota group that fail to demonstrate sexual reproduction within the host despite the presence of a characterized sexual reproductive ability, *A. fumigatus* can in a synthetic setting undergo mating. However, the interaction with the host may have made it unnecessary during the infection process.

#### Pneumocystis carinii

Related to both *S. cerervisiae* and *Sc. pombe*, *Pneumocystis* species are fungal pathogens of the mammalian lung. While previously all were referred to as *P. carinii*, recent reclassification has reserved this particular designation for the species infecting rats (Skalski et al., 2015). Like other ascomycete fungi, this group of infectious fungi displays a very narrow range of host specificity, in so much as human isolates will not establish infection in other mammalian hosts (Skalski et al., 2015). All stages of the lifecycle of this fungus have been found within the host (Chary-Reddy and Graves, 1996), indicating that unlike the previously described mammalian pathogens, mating, if it occurs, would happen inside the host.

The genome of the *Pneumocystis* species is relatively small compared to other related fungi, with a reduced inventory of transcription factors and metabolic enzymes (Ma et al., 2016). Exploration of the genome of *P. carinii* and the human pathogen *Pneumocystis jirovecii* found components of the mating type loci similar to those found in the related ascomycete *Sc. pombe*. Only mating three genes, *matMc*, *matMi*, and *matPi* were found in the genomes of both of these organisms (Almeida et al., 2015). These three genes appeared to be linked together within the genome and no sequences indicating mating type switching were present (Almeida et al., 2015). This finding led authors to hypothesize that homothallism is the system of reproduction for *P. carinii* (Almeida et al., 2015), a system in which a mating partner is not required, since a single organism can produce cells that can mate. Additionally, a putative pheromone receptor gene was found within the genome, orthologous to *ste3* of *Sa. cerevisiae* (Smulian et al., 2001). Other genes related to the pheromone response cascade, such as those for Ste20 protein kinase and Ste12 homeodomain protein, and a potential pheromone precursor, were found in the genomic region encoding this putative receptor, similar to the arrangement of other mating type loci (Smulian et al., 2001). Interestingly, this arrangement of the mating type locus was more similar to that of *Cryptococcus neoformans*, a basidiomycete fungus, rather than to other ascomycetes (Smulian et al., 2001). Although the putative pheromone receptor exhibited the same structural domains and localization to the cell membrane as similar proteins in other species, it did not bind either **a** or α factor from *Sa. cerevisiae* (Vohra et al., 2004).

Despite the lack of evidence of sexual reproduction in *P. carinii*, other indirect evidence suggests it is still possible, though not yet observed. Upon attachment to lung cells, expression of genes canonically involved in ascomycete mating, specifically *ste20*, is highly upregulated (Kottom et al., 2003). Moreover, expression of the *P. carinii* version of *ste20* rescued the mating-related phenotypes in *Sa. cerevisiae* mutants lacking their endogenous Ste20. (Kottom et al., 2003). *P. carinii* does go through a diploid phase and it is hypothesized that mating may occur during the formation of the diploid (Skalski et al., 2015). Unlike other related organisms that may only infrequently mate within the mammalian host, this species gains the benefits of meiotic recombination in the absence of a compatible mating partner by using a homothallic system, yet another strategy of sexual reproduction influenced by ecological niche.

An analysis of the components of the mating type loci in members of the Ascomycota provides an understanding of potential reasons for sexual reproduction to have evolved in fungi (see **Figure 2** for a schematic of representative Ascomycete mating loci). While all members of this group produce the genetic components necessary for sexual reproduction, not all the fungi use these components, and those that do seem to use them infrequently in wild populations. Most likely as a means of DNA repair, sexual reproduction evolved as a method to right the wrongs created by nutrient starvation or exposure to other potentially damaging agents. For strictly saprophytic organisms like *Sa. cerevisiae* and *Sc. pombe*, sexual reproduction still occurs, albeit infrequently, in wild populations and can be induced synthetically through nitrogen starvation. In infectious organisms, the ability to sexually reproduce is even more rarely seen in wild isolates, either due to the loss of fitness as an infecting agent in the case of *Ca. albicans* or due to a loss of necessity of mating in the case of *Mag. grisea* or *F. oxysporum*. The selective pressure in favor of successful infection of a host far outweighs any benefit gained from sexual reproduction. However, as is seen in *F. oxysporum*, the components of the mating pathway remain vital to the fungus, particularly in the case of *F. oxysporum*, as a means of host interaction. An examination of the other members of *Dikarya*, the Basidomycota, provides further evidence of the interconnectivity of sexual reproduction and selective fitness for both non-pathogenic and pathogenic fungal members. Most of these fungi engage in sexual reproduction and in the context of lifestyle, different selective pressures have resulted in evolution within the mating locus and with process of mating.

## The Basidiomycota

The phylum *Basidiomycota* can be divided into three major lineages: mushrooms, rusts and smuts. The most recent attempt at phylogenetic classification based on available molecular data resulted in the formation of three subphyla: *Agaricomycotina* (mushrooms), *Pucciniomycotina* (rusts), and *Ustilaginomycotina* (smuts), although the order of emergence in evolutionary history is unclear (Ebersberger et al., 2012). In many Basidiomycota, the dikaryotic state is maintained by clamp connections that help reestablish the pairs of compatible nuclei after synchronous mitotic divisions. Periodically, the long-lasting dikaryons produce usually club-shaped end cells, the eponymous, basidia. It is here that karyogamy occurs, yielding the diploid cell. Shortly thereafter, meiosis occurs and the resulting four nuclei migrate into four usually external apical cells, the basidiospores. Although basidia are microscopic, they may be produced on or in large multicellular spore-bearing structures (i.e., basidiocarps) such as mushrooms or puffballs (Kirk et al., 2008). The tetrapolar mating system, where both the homeodomain portion of the locus and the pheromone/pheromone receptor portion of the mating type locus are physically unlinked, is unique to this group, although not all members use this system. Analysis of various organisms of this phylum provides further understanding of the function of the mating type loci in the formation and maintenance of each organism’s ecological niche.

While not meant to be encyclopedic, this review will focus both on primarily saprophytic and on obligate or facultative pathogenic species; here we will not describe in detail fungi whose lifestyle is symbiotic. For example, the ectomycorrhizal fungi contain members of the Ascomycota, Basidiomycota, and Zygomycota. These fungi form symbiotic relationships with roots of their host plants (Kirk et al., 2008). In natural conditions, basidiomycete ectomycorrhizal fungi, e.g., *Laccaria bicolor*, are typically in the dikaryotic state when forming symbioses with trees. As representatives of basidiomycete ectomycorrhizal fungi, the *Laccaria* genus, comprising some 75 species, includes some useful experimental models. In particular, *L. laccata* and *L. bicolor*, have been particularly useful, since they can germinate from basidiospores and/or grow vegetatively. Moreover, the *L. bicolor* genome has been sequenced (Martin and Selosse, 2008), and, like the fungi below that are discussed in detail, they mate via a tetrapolar mating system. Like *Coprinopsis cinerea* (discussed below), for *L. bicolor*, the *A* mating type locus contains homeodomain proteins and the *B* mating type locus contains a pheromone and pheromone receptor system. Unlike *Co. cinerea*, *L. bicolor* has a single pair of homeodomain proteins at the *A* locus and much fewer allelic variations (Niculita-Hirzel et al., 2008), giving rise to fewer compatible mating types. Additionally, evolution at the *B* mating locus is not characteristic of other sex-determining genomic regions, with many duplications and transposable elements (Niculita-Hirzel et al., 2008). Potentially this lack of conservation within the *B* locus accounts for the many species within the *Laccaria* genus, as evolution at this locus would be species-specific (Niculita-Hirzel et al., 2008). Notably, *L. bicolor* can be infectious as either a monokaryon or dikaryon, with dikaryotic cultures being the more vigorous colonizers (Kropp et al., 1987). These fungi are also interesting from the perspective that certain stresses and exposure to potential DNA damage are associated with the establishment of the ectomycorrhizal association, at least for some potential hosts (Smith and Read, 2010). Contact of the hyphae with root cells and subsequent digestion through the apoplastic space of *Picea abies* or *Eucalyptus globulus* leads to host production of chitinases and peroxidases that could inhibit formation of the Hartig net associated with symbiosis. Although plant cells also are also up-regulated for stress- and defense-related proteins, the normal massive host cell death associated with hypersensitive response does not occur, and by 21 days after colonization, such host resistance symptoms have diminished to the extent that a compatible interaction is eventually formed. Thus, somehow the fungus is able to suppress this host response, as well as to deal with the stresses associated with establishing these interactions (Smith and Read, 2010).

Two of the major lineages of *Basidiomycota* are pathogens, requiring a host to complete their lifecycle. As the pathogen and the host tend to evolve together, different selective pressures apply to these organisms that saprophytic organisms do not encounter. For these organisms, mating serves as both of mechanism of self versus non-self recognition as well as, in some cases, a means by which to enter the specific host. Increasing host specificity as well as intraspecies recognition are different selective pressures that have shaped the evolution of the mating type loci in these organisms. Human pathogens represent the extreme example within the basidiomycete fungi, where the sexual cycle is not implicated in the infection process.

### Saprophytic Basidiomycota

Generally speaking, saprophytic basidiomycete fungi include mushroom species. Like their ascomycete cousins, these organisms live off dead and decaying organic matter. This group of organisms exemplifies the use of sexual reproduction as a means of increased variation within the fungal kingdom. Most of the species studied have many alleles at multiple mating type loci, creating much more than two compatible mating types. Sexual reproduction within higher eukaryotes is driven by the selective benefit of creation of variation within the population, but is limited to mating between one of two types of parents, for example male and female. Within the mushrooms, many thousands of “sexes” are created by variation within the multiple alleles of the mating type loci.

#### Coprinopsis cinerea

Potentially one of the most well studied mushroom species, *Coprinopsis cinerea*, also called *Coprinus cinereus*, is a saprophytic species of mushrooms, known to be an important ecological decomposer. *Co. cinerea* is well-suited to the study of meiosis because meiosis progresses synchronously within the mushroom cap in roughly 10 million cells, and the prophase stage is prolonged. As in most mushroom systems, mating between compatible mating partners results in the formation of a dikaryon on which mushroom fruiting bodies develop (Asante-Owusu et al., 1996). There are two groups of mating type genes in *Co. cinerea*, *A* mating-type genes and *B* mating-type genes. *A* and *B* independently regulate different steps in dikaryon development and allow self versus non-self recognition (Kües et al., 1992). The *A* mating-type genes encode several genes that can be characterized as providing one of two classes of homeodomain proteins, HD1 or HD2 (Asante-Owusu et al., 1996). Seven *A* genes have been identified, four of which encode homeodomain proteins, and these four genes determine mating type specificity (Kües et al., 1992). In a compatible mating partner, only one of these alleles must be different between the two partners and one heteroallelic combination in the mated pair is sufficient to trigger *A*-regulated development (Kües et al., 1992). Different combinations of these four *A*-specific alleles generate the many different mating types in nature (Kües et al., 1992).

Compatible mating partners bring together different versions of homeodomain proteins that can heterodimerize, generating active transcription factors that lead to sexual development (Asante-Owusu et al., 1996). The heterodimerization mediated by the N terminus of these homeodomain proteins is an essential component of self versus non-self recognition (Asante-Owusu et al., 1996). Different *A* genes seem to be responsible for clamp cell formation (Asante-Owusu et al., 1996). In addition to this aspect of mating partner recognition, the *B* mating locus encodes pheromones and pheromone receptors that also serve in identification of potential mates and govern adjacent cell fusion during mating (Asante-Owusu et al., 1996). The *B* mating locus encodes peptide pheromones and corresponding 7-transmembrane helix receptors for their recognition (O’Shea et al., 1998). A single *B* mating type locus may contain genes for as many as three receptors and six pheromone precursors (O’Shea et al., 1998). As with the *A* mating locus, a single different pheromone precursor gene or pheromone receptor gene in another cell is sufficient for mating compatibility (O’Shea et al., 1998), as the pheromone receptor can recognize any other pheromone produced from a different *B* sublocus as being “non-self.”

This mushroom species clearly has benefitted from the generation of variability through sexual reproduction. Due to the multiallelic state of both the pheromone and pheromone receptor-containing locus as well as the homeodomain protein-containing locus, this system generates a potential for outcrossing of greater than 50% (O’Shea et al., 1998). In nature, this translates into both the increased probability of encountering a compatible mating partner as well as the generation of great genetic variability within a population due to the numerous possibilities of outcrossing. For this organism, sexual reproduction is an essential element of niche formation by increasing fitness through successful mating. Also of note, genes induced in this species by meiosis are more highly conserved than genes not induced by meiosis in ascomycete fungi like *Sa. cerevisiae* and *Sc. pombe*, despite the divergence of these species between 500 and 900 million years ago (Burns et al., 2010), indicating the clear conservation and importance of meiosis within the fungal kingdom. Examination of another mushroom species indicates just how many different mating types can be generated with multiallelic mating loci.

#### Schizophyllum commune

Another well studied mushroom, *Schizophyllum commune*, is found in many diverse environments and typically as a wood rot fungus or occasional pathogen of woody species (Ohm et al., 2010). While this fungus has recently been found as an opportunist in human infections of immunocompromised hosts, causing, for example, lung infections, and has been associated with soft tissue and nail infections in other animals (Chowdhary et al., 2013) for the most part it is considered as a saprobe and thus, we deal with it here as such. Like *Co. cinerea*, *Sch. commune* has two multiallelic mating locus regions. The *A* mating type region, containing *A*α or *A*β regions, encodes homeodomain proteins to regulate *A*-dependent sexual development. There are at least three *A*α mating types, Aα1, Aα3, and Aα4. A*α3* and A*α4* both encode two polypeptides, Y and Z, while *Aα1* encodes a single polypeptide Y (Stankis et al., 1992). The Z and Y polypeptides are homeodomain proteins, and the Y product also has a putative DNA binding domain (Stankis et al., 1992). In a compatible mating pair, the Y from one partner interacts with the Z from another partner to regulate Aα-regulated development (Stankis et al., 1992). The *A*β loci are functionally redundant with the *A*α loci, and there have been at least 32 different variations of *A*β loci identified in wild populations (Specht, 1995).

The *B* mating type region also contains two tightly linked loci, *B*α and *B*β, and, like their *A* counterparts, they are functionally redundant, both encoding pheromone and pheromone receptors that govern self vs. non-self recognition (Wendland et al., 1995). While the two loci are functionally redundant, receptors from *B*β do not respond to *B*α pheromones (Wendland et al., 1995). Differences at either *B*α or *B*β between a mated pair of individuals is sufficient to initiate *B*-dependent sexual development (Vaillancourt et al., 1997). An analysis of the *Bα1* locus found it contains three pheromone precursors and one pheromone receptor (Vaillancourt et al., 1997). The *Bβ1* locus also contains the genes that function with *B*β specificity. Comparison of the DNA sequence of *Bα1* and *Bβ1* suggest they may be the result of a duplication event (Vaillancourt et al., 1997).

As with the *Co. cinerea* system, the *A* and *B* mating loci are responsible for different parts of sexual development through transcriptional regulation. *A*-dependent regulation results in the upregulation of genes having to do with the cell cycle and the down regulation of genes related to metabolism, while *B*-dependent genes include those for cell wall and membrane metabolism, stress response, and the redox state of the cell (Erdmann et al., 2012). Because of the redundancy of function of *A*α and *A*β genes, as well as *B*α and *B*β genes, a compatible mating partner does not have to have different alleles at all four loci. At least 28,000 different mating types of *Sch. commune* exist because of the different number of alleles at these four loci, and any individual mating type can mate with all but three of the others (Specht, 1995). Selective pressure on this system has strongly favored increased variation through mating and diversity at the mating type loci to increase the possible mating partners available.

While the mushroom species have acquired large numbers of allelic variation within the mating type loci, other basidiomycete fungi have very little variation within these loci across the population, indicating very little out crossing occurs in the population. Like the ascomycete fungi, the basidiomycete human pathogens tend to avoid mating in clinical populations, while they retain the genetic components required for mating. Other members of this group that mate, do so with other haploid cells from the same meiotic division, and while this strategy does not increase genetic diversity in the population by the mixture of genetic information between two unrelated haploid cells, it does guarantee that the fungus never has to leave the comfort of its host plant to find a compatible mating partner. Still other Basidiomycetes require a compatible, unrelated mating partner to complete their life cycle and enter their respective host. It is of note that despite the variety of mating strategies, the types of organism that engage in each seems to be relatively consistent. For example, while human pathogens avoid mating, saprophytic organisms have accumulated variation at the mating type loci and in turn several thousand “genders.”

### Parasitic Basidiomycota

Like their ascomycete cousins, basidiomycete pathogens face a different set of selective pressures than closely related saprophytic organisms due to their interaction with a host. In a subset of organisms that are phytopathogens, mating is essential for host penetration, meaning meiosis remains an essential element of the lifecycle as the haploid cells capable of mating are the result of meiosis of a diploid cell. This is in contrast to many of the strategies developed by ascomycete plant pathogens that have ceased to use their ability to mate as it is not required to enter the host plant. However, like the ascomycete mammalian pathogens, basidiomycete fungi that infect human hosts have elusive or absent meiotic and mating pathways despite the genetic components necessary for both.

Unlike saprophytic organisms, parasites must also be able to withstand the defenses of the host. Reactive oxygen species are often produced by plants in response to fungal invasion, which can do damage to fungal genomic integrity. Enzymes involved in eliminating these damaging molecules have been found to be essential virulence factors in many microbial pathogens of plants and in many cases, the invading pathogen uses this defense mechanism against the plant (Kim, 2014). In these phytopathogens, the occurrence of meiosis following plant infection allows for repair of any DNA damage that may have occurred as a result. A number of mushroom species have been described as pathogens (for a review, see de Mattos-Shipley et al., 2016). For example, *Moniliophthora perniciosa*, the causal agent of witches’ broom disease of cacao, a hemibiotroph, has a typical basidiomycete lifestyle and morphology, forming clamp connections and producing mushrooms (de Mattos-Shipley et al., 2016). Additionally, *Armillaria mellea*, also a plant pathogen, causes *Armillaria* root rot in many plant species and produces mushrooms around the base of trees it has infected (de Mattos-Shipley et al., 2016). Nevertheless, we will not discuss the mating programs and meiosis for these organisms since, for example, *Armillaria mellea* appears to have a persistent vegetative diploid state in nature (Anderson and Ullrich, 1982) and the mating system appears to be similar to that of *Co. cinerea* (Ullrich and Anderson, 1988). Similarly, *M. perniciosa* exhibits primary homothallism as its reproductive strategy; thus, the change from the monokaryotic to the dikaryotic mycelium occurs without the prerequisite of mating between compatible individuals (Mondego et al., 2008). Accordingly, in the sections that follow, we focus on well-characterized mating system examples from other phytopathogenic fungi, especially smuts, as well as the mostly opportunistic human pathogen, *Cryptococcus neoformans*.

#### Ustilago maydis

**FIGURE 3:**
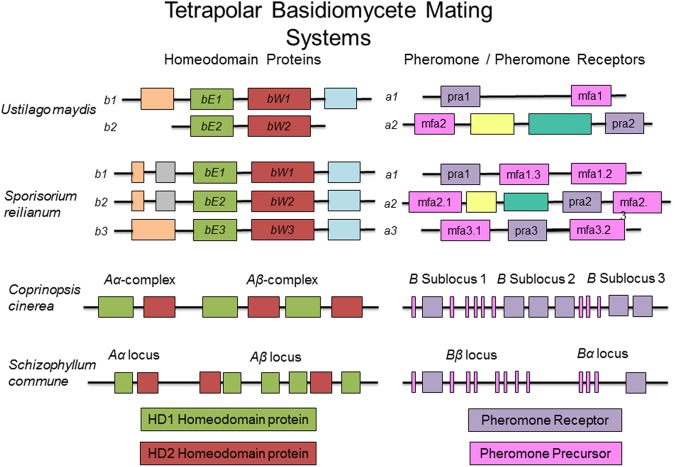
Sample tetrapolar mating systems in basidiomycete fungi. While the number of homeodomain proteins may vary across this group, the composition of genetic material at the mating type loci is conserved. Boxes of the same color represent orthologous components in the different mating types and across species.

**FIGURE 4:**
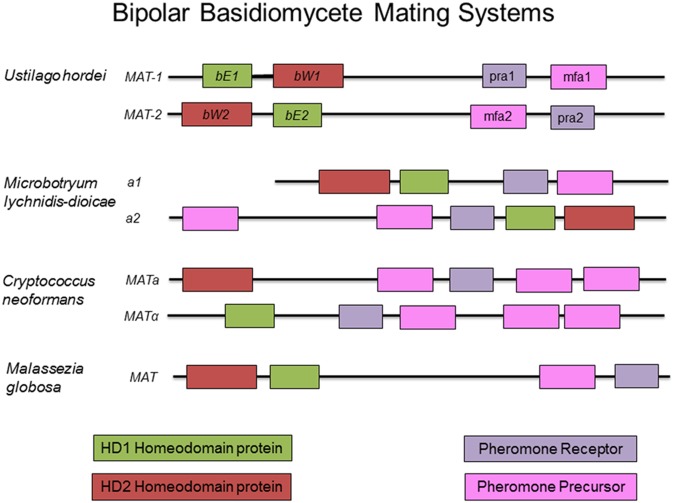
Sample bipolar mating systems in basidiomycete fungi. While the two mating type loci of these organisms have become physically linked by chromosomal arrangement, the composition of the mating type loci remains similar to that found in tetrapolar systems. Boxes of the same color represent orthologous components in the different mating types and across species.

The fungal tree of life remains a dynamic entity, particularly with the increased availability of molecular and genomic data. While there is some uncertainly as to the exact placement of many plant pathogens within a fungal phylogenetic tree, the conservation of many aspects of the mating type locus remains indisputable. *Ustilago maydis*, the causative agent of corn smut, is potentially the most well studied member of the Basidiomycota, and many would argue a better model organism for eukaryotes than *Sa. cerevisiae*. It has become the standard against which other mating type loci of this group are analyzed (see **Figures 3**, **4** for a comparison of basidiomycete mating type loci). In nature, *U. maydis* is found as haploid sporidia. When sporidia of opposite mating type recognize each other, they form conjugation tubes and the resulting dikaryon is able to penetrate the host plant. Inside the plant, numerous diploid teliospores are formed inside galls or tumors, giving rise to the characteristic smutted appearance of the plant. Under suitable conditions, these teliospores germinate, producing haploid sporidia and the cycle continues.

The mating type locus in *U. maydis* has been studied extensively and includes two genomic regions on different chromosomes (**Figure 3**). The *a* mating type locus of *U. maydis* encodes the pheromone and pheromone receptor system. The allele at the *a* locus is identified as either a1 or a2. Molecular analysis of the *a* alleles reveals that the a1 allele is smaller, at 4.5 kb while the a2 allele is larger at 8 kb (Bolker et al., 1992). The sequences show no homology to each other and are absent in strains of the opposite mating type; i.e., the a1 allele is specific to a1 strains (Bolker et al., 1992). Thus, technically they are not true alleles and, as was seen in *Sa. cerevisiae*, the two different forms are commonly referred to as idiomorphs (Metzenberg and Glass, 1990). The genes *mfa1* and *mfa2* encode pheromone precursors in their respective mating types and are the only pheromone precursors. Mutants deleted for *mfa1*, for example, are unable to fuse to haploid cells of the opposite mating type (Bolker et al., 1992). The pheromone receptors are encoded by the *pra1* and *pra2* genes, and are very similar to the *Sa. cerevisiae STE3* receptor gene (Bolker et al., 1992). The receptors are located on the cell surface and bind to a secreted pheromone from a cell of the opposite mating type, resulting in a cellular response leading to preparation for mating (Bolker et al., 1992).

The *b* locus of *U. maydis* contains two unrelated homeodomain proteins that are most different in the N terminal region (Kamper et al., 1995). The two proteins are either b-East (bE) or b-West (bW), and there have been at least 25 different alleles identified from wild isolates (Kronstad and Leong, 1990). Deletion of a gene encoding a single homeodomain protein has no effect on phenotype (Gillissen et al., 1992), suggesting a redundancy of function. Self/non-self recognition is mediated by dimerization of two homeodomain proteins from different mating types, for example bE1 with bW2 (Kamper et al., 1995). The heterodimer acts as a transcription factor for genes necessary to establish the infectious dikaryon and proliferate within the host (Kronstad and Leong, 1989). Any combination of different alleles from the *b* locus can cause the dimorphic switch from yeast-like to infectious filamentation and trigger the pathogenic program (Schulz et al., 1990). It is possible to form haploid infectious strains simply by introducing homeodomain proteins at the *b* locus that are capable of forming a heterodimer (Kronstad and Leong, 1989). While in nature, b proteins derived from the same allele cannot dimerize, synthetic fusions of bE2 and bW2, for example, result in pathogenic development when introduced into haploid strains deleted for the native *b* locus (Romeis et al., 1997).

**FIGURE 5:**
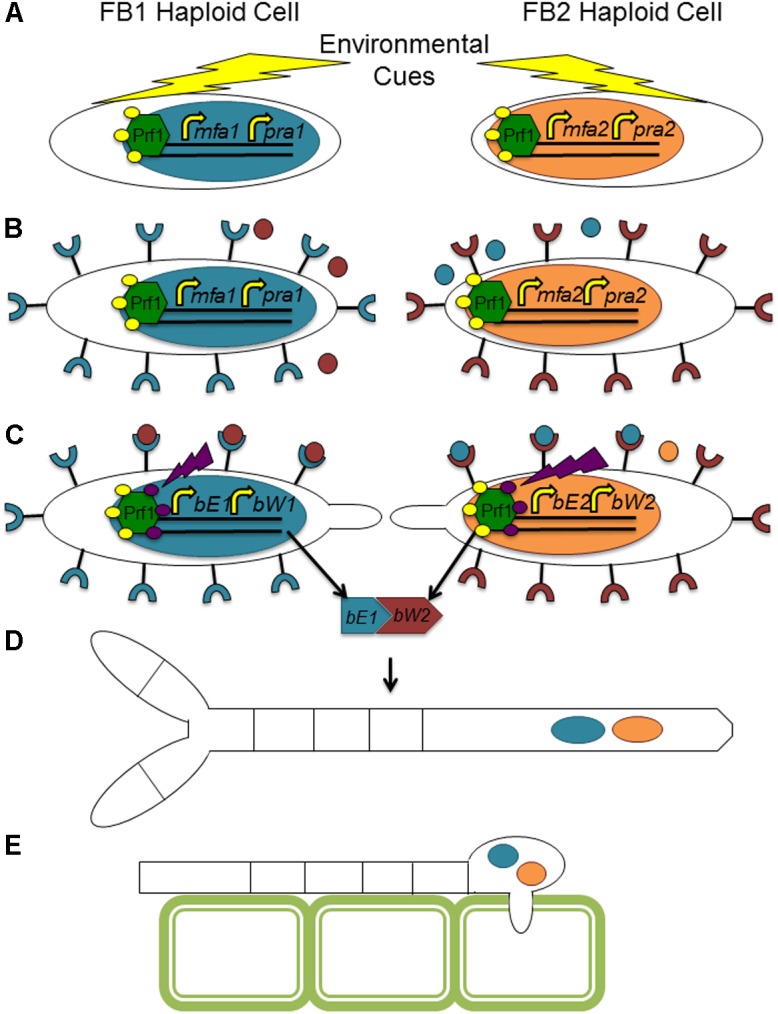
Mating in *Ustilago maydis*. **(A)** An initial environmental cue, which may be from the plant or involve nutrient limitation, results in the initial phosphorylation of Prf1. **(B)** Prf1 binds to a response element in the *a* mating type loci, resulting in increased expression of the pheromone and pheromone receptor in both types of haploid cells. **(C)** Interaction of the pheromone with the receptor on the cell of opposite mating type results in additional phosphorylation of Prf1. Prf1 phosphorylated by both pathways then binds to response elements within the *b* mating type locus, leading to increased expression of the homeodomain proteins. Additionally, interaction of pheromone and receptor results in the growth of conjugation tubes from each cell toward each other. **(D)** Creation of a homeodomain heterodimer comprised of *bE* and *bW* from different allelles results in the formation of an infection dikaryon. **(E)** This dikaryon is able to establish infection within the host, although fusion of the nuclei does not occur during infection until teliospore formation, just prior to release from the infected plant galls.

Compatible mating strains recognize each other through the pheromone and pheromone receptor system. For example, after modification, the pheromone Mfa1 secreted by a1 cells is recognized by a pheromone receptor, Pra2, found in the cell membrane of a1 cells. As with *Sa. cerevisiae*, pheromone binding to receptor leads to arrest in cell cycle, although for *U. maydis*, the arrest is in G2 (García-Muse et al., 2003). Upon interaction of the pheromone with its receptor, a signaling cascade is activated that leads to phosphorylation of a transcription factor, Prf1 (Hartmann et al., 1996). Prf1 binds specifically to pheromone response elements within the *a* and *b* mating loci, increasing transcription from both mating type loci and is required for pathogenic development (Hartmann et al., 1996). In addition to the activation of Prf1, exposure of compatible mating partners to pheromone results in the growth of conjugation tubes growing toward each other from each haploid cell and eventually fusing (Snetselaar and Mims, 1992; Banuett and Herskowitz, 1995). This fusion results in the formation of a dikaryon, and if the two nuclei within this dikaryon also have different alleles at the *b* mating type loci, filamentation is induced and this structure is able to infect the host corn plants (**Figure 5**).

The *b* locus seems to be more involved in the development of pathogenicity than the *a* locus, as diploids that are heterozygous for *a* but homozygous for *b* grow in a yeast-like manner and fail to establish infection in host plants (Schlesinger et al., 1997). Heterozygosity at the *a* locus is not required for pathogenicity, and diploids homozygous for *a* but heterozygous for *b* form similar tumors in host plants as mated haploid strains. However, in nature, the function of both the *a* locus and the *b* locus in a multiallelic incompatibility system functions to limit inbreeding and increase variability within the population (Yee and Kronstad, 1993). Also, in the synthetic setting of the lab, organisms must be deprived of nutrients, specifically ammonium and possibly phosphorous, in order to see the mating phenotype. Mating is typically observed for *U. maydis* and *U. hordei* on activated charcoal medium as it sequesters inhibitors of mating, including nutrients (Martinez-Espinoza, 1993). Similarly, in nature, mating occurs during appropriate environmental conditions. In this system, mating and sexual reproduction are vital to plant infection. Only synthetically made laboratory haploid strains are able to infect the host corn plant, and in nature, mating and formation of the infectious dikaryon is paramount to establishing infection inside the host, gaining access to the resources inside the host, and proliferating the species. Because of the intrinsic connection between nutrient limitation and induction of mating, it is likely that invasion of the host gives the organism access to nutrients that are needed for survival. Successfully accessing necessary nutritional components by invading the plant can only be accomplished by being able to recognize a suitable mate, mediated by the mating type loci. On the other hand, within the host, *U. maydis* must further manipulate the host by reallocating organic nitrogen sources within the plant to turn the tumor into a “nitrogen sink” (Horst et al., 2010).

In *U. maydis*, there are only two *a* mating type locus idiomorphs, *a1* and *a2*. However, with more than 25 variations at the *b* mating type locus, there is still the possibility of more than two “sexes” that are compatible mating partners. Although the number of different compatible mating strains is much smaller than is observed in the mushrooms, outcrossing does seem to be the preference among this species as well. While the two different haploid cells generated by the germinating teliospore theoretically should be compatible mating partners, mating between these haploids does not appear to occur often in nature. Examination of two geographically isolated populations of wild *U. maydis* revealed that *U. maydis* populations in both areas generally arose as a result of outcrossing, not mating between products of the same meiosis event (Barnes et al., 2004). Because mating is a requirement for host entry in this system, the same potential cost of losing host-specific genetic information is not a driving force in the maintenance of sexual reproduction in this system. While phytopathogenic ascomycete fungi enter their hosts as haploids and the examples reviewed here rarely mate in wild populations, since *U. maydis* must begin the mating process prior to host entry, the chance of the loss of host-specific genetic information from shuffling during meiosis is unlikely. As such, the benefit of variation within the population gained from outcrossing seems to be a stronger evolutionary pressure. Examination of another closely related species reveals how slight modifications to the maturation process of mating competent cells can further guarantee the recognition of the correct compatible mating partner.

#### Sporisorium reilianum

Another smut fungus similar to *U. maydis*, *Sporisorium reilianum*, exists in two varieties based on host specificity, one that infects maize (SRZ) and one that infects sorghum (SRS) (Zuther et al., 2012). Like some other closely related smuts, the mating type locus is composed of two unlinked genomic regions, *a* and *b*, which code for a pheromone and pheromone receptor and homeodomain proteins, respectively. The genetic material within and around these loci shows a high degree of synteny with *U. maydis* (Schirawski et al., 2005). The *b* locus exists in at least five different alleles and encodes two subunits of a heterodimeric homeodomain transcription factor (Schirawski et al., 2005). In contrast to *U. maydis* which has only two idiomorphs, the *a* locus of *Sp. reilianum* can be one of three alleles, each containing two pheromone precursors (Schirawski et al., 2005). These different versions, designated a1, a2, and a3 are idiomorphs. Each idiomorph encodes different pheromones and pheromone receptors. The pheromones for a particular receptor have identical sequence even if they exist in a different allele; that is, the pheromones from a1 and a3 that bind to the receptor from a2 have the same sequence (Schirawski et al., 2005). Two of these pheromones, those binding to the receptors of a1 and a2, show a high degree of homology to the corresponding genes in *U. maydis*; the third set, binding to the receptor from a3, while they are identical to each other, are only weakly related to other fungal pheromone precursors (Schirawski et al., 2005). This arrangement of two pheromone precursors and one pheromone receptor in each *a* idiomorph appears to have arisen from a recombination event within the locus itself and serves to increase the number of mating types within the species (Schirawski et al., 2005).

Interestingly enough, despite the narrow host range of the two varieties of *Sp. reilianum*, there is remarkable similarity between the pheromones of the two (Radwan, 2013), and therefore the possibility that the two subspecies could potentially mate in natural populations. However, despite the similarity of the genetic material within the mating type loci, SRS is only successfully able to produce teliospores within sorghum and SRZ is only able to do the same in maize. In its respective host species, each is able to resist the attempts of the plant to clear the infection, while in the opposite species, the fungus succumbs to the plant’s defense mechanisms. In this case, selection has most likely not favored variation within the mating type locus to reduce promiscuity between different species but instead has favored the maintenance of other specific genes that allow proliferation within the host despite the plant’s attempt to rid the infection. Like other basidiomycete fungi, such as the mushrooms discussed above, more allelic versions of the *a* locus allow for more mating types and greater variation within the natural populations. Successful mating only within appropriate hosts guarantees that offspring only carry parental DNA from organisms that are able to establish infection in the host.

#### Ustilago hordei

Recent molecular genomic analysis has placed *Ustilago hordei* and *Ustilago maydis* as more distant relatives than their genus name would suggest (McTaggart et al., 2016). However, between these two species, the conservation of genetic material within the mating type locus is undeniable. Just as *Sa. cerevisiae* serves at the standard for ascomycete fungi, *U. maydis* may serve as one model organism for the Basidiomycota. The elements and arrangement of components of both the *a* and *b* mating type loci are highly conserved in their sequences, although the arrangement of genes varies greatly within the group, *U. hordei* infects barley and like *U. maydis*, forms large masses of black teliospores within the host plant. Like all smuts, the two fungal pathogens share many life cycle features, including a yeast-like stage and an infectious dikaryon arising from the fusion of two haploid cells (Bakkeren et al., 1992). Unlike the *U. maydis* mating system which is tetrapolar, the *a* and *b* loci being on different chromosomes, *U. hordei* has a bipolar mating system in which the *a* and *b* loci are physically linked on the same chromosome and designated as one of two mating types, MAT-1 or MAT-2 (Bakkeren and Kronstad, 1993) (**Figure 4**). In the *U. hordei* bipolar system, every fusion event mediated by the *a* locus will guarantee that the *b* locus will also be of opposite mating type and the resulting fusion will form an infectious dikaryon (Bakkeren and Kronstad, 1994).

Like the *U. maydis* mating locus, the *a* locus in *U. hordei* encodes the pheromone precursors and pheromone receptor genes, with a high degree of similarity to the sequences contained within the *U. maydis* locus (Bakkeren and Kronstad, 1994). Also similar to the other smut, the *b* locus encodes two homeodomain proteins that during mating dimerize to create a transcription factor to regulate expression of mating-related genes (Bakkeren et al., 2008). Interestingly, there is a high degree of sequence similarity between the *U. hordei* mating type locus and *MAT* loci of other fungi, including *U. maydis* and *Sa. cerevisiae* (Bakkeren et al., 1992). In fact, if the homeodomain of “opposite mating type” from *U. maydis* is expressed in *U. hordei*, and vice versa, there is a positive mating reaction on charcoal plates, although pathogenicity in corn is markedly reduced, i.e., no teliospores are produced and only weak symptoms of infection appear (Bakkeren and Kronstad, 1996). Analyzing the morphological changes that occur upon induction of the mating reaction in *U. hordei*, it seems that to ensure mating with a member of the same species, the conjugation tubes of this fungus develop at a later time than those of *U. maydis*, for example.; Thus, even if the two different species were to encounter each other, mating is unlikely to occur because of the timing of the conjugation tube formation and as such metabolic energy would not be wasted on a fruitless venture (Bakkeren et al., 2006).

Another interesting characteristic of the *U. hordei* mating type locus is the similarities it shares with sex chromosomes. While the mating type locus is located within a chromosome that encodes unrelated processes, it is unusually large compared to other smuts and has an accumulation of repetitive DNA and retrotransposons, few of which are found in *U. maydis* (Bakkeren et al., 2006). In addition to the presence of genes for pheromone precursors, pheromone receptors and homeodomain proteins regulating mating and virulence, the *U. hordei MAT* locus also contains 47 other genes, most of which have homologs in *U. maydis* but are not physically linked to the mating type locus (Bakkeren et al., 2006). Because the mating type locus in *U. hordei* brings along much of the necessary genetic information for successful mating, variability in this region has not been favored by selection; and, by having a specially timed mating program, this fungus has become quite specialized to parasitizing cereal grasses (Bakkeren and Kronstad, 1994). Although *U. hordei* lacks the variation in the mating type locus seen in other closely related smuts, because encountering a compatible mating partner guarantees successful mating due to the physical linkage of the pheromone/pheromone receptor complex and the homeodomain proteins, as well as the unique timing of the formation of conjugation tubes in this organism, variability is not necessary for successful parasitism within the group and linkage of other genes required for infection to the mating locus ensures that they segregate in a way that is favorable to allow the fungus to gain access to its host. Examination of another basidiomycete biotrophic fungus reveals that host specificity is mediated by the organism’s ability to respond to the host’s immune response, and that while interspecies mating may occur, only mates from compatible haploids of the same species result in teliospore formation on the host and successful passage of genetic material to future generations.

#### Microbotryum lychnidis-dioicae

Previously called *Ustilago violaceum* and *Microbotryum violaceum* (Day, 1979; Hood and Antonovics, 1998, 2000; Hood et al., 2013), *Microbotryum lychnidis-dioicae* is a member of the *Microbotryum violaceum* species complex or anther smuts (Hood et al., 2013), which is found in the subphylum *Pucciniomycotina. Mi. lychnidis-dioicae* is the causative agent of anther smut on *Silene latifolia*. This fungus is a relatively unique system to study because it acts as a sexually transmitted disease and is capable of changing the gender of its host. This fungus infects its host and replaces the pollen on anthers with diploid teliospores that are then carried to a new host by bees and other pollinators. Once on the new host, the teliospores germinate, undergo meiosis, and produce haploid sporidia. If sporidia of compatible mating type encounter each other, they form conjugation tubes and mate, producing dikaryotic hyphae that can grow inside plant tissue. The infection becomes systemic, eventually leading to the production of teliospores in the place of pollen and the cycle continues (Giraud et al., 2008). Like *U. hordei* and *Sp. reilianum*, *Mi. lychnidis-dioicae* guarantees successful mating on an appropriate host plant, but the strategy is unqiue.

The mating type loci of *Mi. lychnidis-dioicae* is the first example of sex chromosomes encountered in fungi (Hood, 2002). The two mating types of *Mi. lychnidis-dioicae*, A1 and A2, are both defined by the different alleles at loci for homeodomain proteins and for pheromone/pheromone receptor systems. The two mating type chromosomes show divergence over 90% of the length flanked by pseudoautosomal regions on either end (Hood et al., 2013). The sex chromosomes are among the largest in this organism’s genome, with A2 being larger than A1 (Hood, 2002). A2 has two genes encoding pheromone precursors, while A1 contains a single locus (Badouin et al., 2015). While the two chromosomes seem to be the result of a duplication event by the number of syntenic blocks, extensive rearrangements in the form of inversions have occurred resulting in the gene order currently present (Badouin et al., 2015). There is significant repression of recombination in the mating chromosomes, resulting in linkage of the pheromone/pheromone receptors and homeodomain protein-encoding genes (Badouin et al., 2015).

Before the nature of the genetic material governing compatible mating partners in this organism was fully elucidated, it was discovered that different environmental factors, including low temperature and nutrient availability activated mating type alleles. As regulators of the developmental switch between three pathways of development, vegetative budding, conjugation, and sexual differentiation, mating type allele activity results in different responses to different environmental conditions. In high temperatures and nutrient levels, cells of this fungus bud vegetatively; however, in correct environmental conditions and upon exposure to the products of cells of opposite mating type (presumably pheromones), cells of a single mating type become blocked in G1 and develop conjugation tubes (Day, 1979). Cells carrying both mating type alleles exposed to similar environmental conditions also could not exit G1 and differentiated into spores (Day, 1979).

Later research also indicated that not only did environmental conditions activate mating, but also governed how the products of mating would act. The promycelium is the product of teliospore germination in this fungus. Generally speaking, the promycelium contains three cells but at low nutrient and temperature conditions, it may only contain two (Hood and Antonovics, 1998). An extensive study of promycelia under different environmental conditions revealed different results based on nutrient availability and temperature. After the first meiotic event within the promycelia, the daughter nuclei are separated immediately by septation. After the second meiotic division, one of the proximal nuclei migrates back into the teliospore; however, based on nutrient availability, the fate of the two distal nuclei can be different (Hood and Antonovics, 1998). At ideal temperature and nutrient levels, the two distal nuclei are separated by a second septation event, resulting in a three celled promycelium, each of which is uninucleated. At low temperature and nutrient availability, the second septation event does not occur, yielding a two-celled promycelium, with one single nucleated cell and one cell with two nuclei; both of these latter nuclei are of the same mating type, as a result of the two components of the mating type locus being linked (Hood and Antonovics, 1998). As the number of heterokaryons that can be formed from a two-celled promycelium versus a three-celled promycelium is different, this environmental response allows flexibility of the mating system to either produce more heterokaryons or more infectious units (Hood and Antonovics, 1998).

Because of the complete linkage of the two components of the mating locus, mating between products of the first meiotic division is possible (Hood and Antonovics, 2000), which ensures the flexibility of the response in promycelia of different number of cells. As such, the mating system of *Mi. lychnidis-dioicae* tends toward mating within the tetrad, ultimately reducing variation in the population and maintenance of deleterious alleles, with rare outcrossing events (Hood and Antonovics, 2000). This rapid intratetrad mating allows the organism to form infectious units to gain access to the host plant in response to environmental conditions such as low nutrient availability without having to encounter another cell of different mating type on the surface of the plant. Outcrossing in this organism seems to be less of an evolutionary driving force; rather continual access to the host plant and avoidance of alleles that would be lethal if given the chance to be expressed in budding haploid cells would here appear to have selected for intratetrad mating as the preferred option (Hood and Antonovics, 2000). At the opposite extreme, are other basidiomycete fungi that also have highly reorganized mating type loci but are rarely seen to mate in natural settings. It is of note that most of the examples of this type of organism are human pathogens, as previous evidence from the Ascomycota demonstrates that some of the products of mating can cause strong immune responses, indicating that avoiding mating may be a better strategy for human pathogens.

### Cryptococcus neoformans

Upon nitrogen starvation or desiccation, the human fungal pathogen *Cryptococcus neoformans* undergoes mating or fruiting, both processes involving meiosis (Lin et al., 2005). The causative agent of fungal infections in both healthy and immunocompromised individuals, *Cr. neoformans*, when present in humans, is primarily a resident of the lung, and it is the largest source of fungal meningitis worldwide. The infectious particle is either a basidiospore or a desiccated cell, small enough to travel deep within the crevices of the human lung (Velagapudi et al., 2009). While sexual reproduction for this organism does not occur within the host, it has been observed. Under suitable environmental conditions, fusion between cells of opposite mating type occurs, producing dikaryotic hyphae characteristic of the Basidiomycota (Wickes et al., 1996). Mating type, either a or α, is defined by the genetic components of the mating type locus in this organism’s bipolar mating system (Kwon-Chung, 1976). Several pheromone precursors and pheromone receptor genes have been identified in the genome but unlike model fungal genomes, such as those from *U. maydis*, these genes are not tightly linked but instead are dispersed throughout the mating type locus (Lengeler et al., 2002). A novel homeodomain protein has been identified within the α mating type alleles, and this novel protein does show a high degree of homology with the *b* locus of *U. maydis* and *U. hordei* (Lengeler et al., 2002). The complementary homeodomain protein was later discovered within the *a* mating type locus and has been demonstrated to interact with the protein from the α mating type locus to regulate mating genes (Hull et al., 2005). Other orthologs of mating-related genes have been identified within the α mating type locus that are also dispersed through a large genomic region (Karos et al., 2000). The evolution of the α mating type locus appears to be similar to that of mammalian sex chromosomes, involving large chromosomal rearrangements and recombination suppression, resulting in a large sex-determining region of generally unlinked genes (Fraser et al., 2004). This may represent yet another transition in the direction of sex chromosomes rather than mating type loci within the genome.

While there is no physiological difference between a and α cells, they do behave differently and are represented in different proportions within clinical isolates (Wickes et al., 1996). Almost all clinical isolates are α mating type (Wickes et al., 1996; Brefort et al., 2005). Both mating types undergo haploid filamentation resulting in the production of infectious spores, although the α cells are more likely to engage in this process (Tscharke et al., 2003). While this organism is capable of sexual reproduction, the independent evolution of mating loci within the two mating types has resulted in one mating type of the organism that can establish infection independently of the other. In fact, mating between two α mating type cells has been observed, to the extent that meiosis and recombination occur (Lin et al., 2005). This phenomenon occurs during haploid fruiting and shares many characteristics of mating, such as the fusion of haploid nuclei and meiosis (Lin et al., 2005). It may offer further insight into why the majority of clinical isolates are of the same mating type. Like *Pneumocystis carinii*, another human fungal pathogen, *Cr. neoformans* may be undergoing a haploid to diploid change at some point in its lifecycle that provides an advantage for infection and virulence (Lin et al., 2005). In this way, within the host, the organism reaps all the benefits of sexual reproduction, such as repair to DNA damage caused by the host response, without the bother of finding a compatible mating partner. This evolution of the mating type locus represents an extreme example of the accumulation of components of canonical mating type components to the extent that all the benefits of mating can be obtained, while creating a nearly clonal population. Although the evolution of the mating type locus has not been driven by host interaction, the lack of necessity for metabolically costly mating has shaped the clinical populations.

### Malassezia globosa

A resident of the human host, typically associated with dandruff, *Malassezia globosa* is closely related to *Ustilago maydis*. This organism and other closely related *Malassezia* species are part of the normal human epidermal flora but some may become infectious during periods of dense growth that lead to the human symptoms of dandruff and seborrheic dermatitis. *Mal. globosa* has a small genome compared to other free living fungi, with only 9 megabase pairs, and appears to be haploid because there are very few polymorphisms within the genome (Xu et al., 2007). The genome contains components similar to the *a* and *b* loci of *U. maydis*, with an arrangement more like that of *U. hordei*, where the genes encoding the pheromone/pheromone receptor system and the homeodomain proteins are physically linked to each other (Xu et al., 2007). Additionally, several orthologs for components of the MAPK pathway in *Sa. cerevisiae* have been identified (Xu et al., 2007). While no sexual cycle has been observed, there are several reasons to believe that this organism is capable of sexual reproduction, including the isolation of strains that appear to be hybrids of other known strains (Saunders et al., 2012). Sexual reproduction in a human pathogen, if discovered, would be unique to this organism, as others, such as *Ca. albicans*, do not reproduce sexually within the host because of the immune response to the products of mating. In contrast to the human pathogens that retain the components necessary for mating but seem to avoid outcrossing, other basidiomycete fungi, particularly the mushrooms have evolved a multitude of variations at the mating type loci for the purpose of creating diversity within the mating population.

## Conclusion

Sexual reproduction, particularly in higher eukaryotes, is the only means of producing additional generations and allows for allelic shuffling between the mating pair. Although this increase in variation seems like it would be favored by natural selection, examination of organisms with sexual and asexual forms of reproduction indicates that the favorability increased variation rendered by sexual reproduction must be weighed against the cost of reproduction, both in the context of cost to the individual organism and, for those organisms that are pathogens, ability to avoid the immune response of a potential host.

Within the Ascomycota, although many species readily mate in the lab, mating in wild type populations is much less common. Although all members of this group seem to possess the genetic information comprising a mating type locus, few regularly engage in sexual reproduction. In saprophytic species like *Sa. cerevisiae* and *Sc. pombe*, while haploid cells of each organism have the genetic information at the mating type loci to make with a compatible mating partner, sexual reproduction is not the default lifestyle of the two, as in wild populations few instances of outcrossing can be found. However, as it can be demonstrated in the lab setting that nutrient limitation can induce meiosis and sporulation, as in the case of *Sa. cerevisiae*, or mating, as is the case for *Sc. pombe*, it is likely that the ability to sexually reproduce has been maintained because organisms with this capability would have greater fitness in the event of adverse environmental conditions. Losing the genetic material at the mating type loci would equate to losing the ability to repair DNA through recombination during mating.

The importance of maintaining the components for sexual reproduction is even more evident in human pathogens like *Ca. albicans*. These organisms only undergo a parasexual cycle, so while they do not outcross often, genetic recombination does occur. Most clinical isolates are white, while mating competent strains are opaque. The switch between these types is governed by the mating type loci. *Ca. albicans* is usually is found as a diploid, heterozygous at the mating type locus; these diploid cells evoke less of an immune response. Because the product of mating, the haploid yeast cells, evoke the strongest immune response from the human host, this organism gets the repair mechanism of mating without expending the cost of potentially alerting the immune system to its presence. Other pathogenic species, particularly plant pathogens, also have developed alternate methods of using the genetic material involved in mating (or not using it at all). The most adaptive example is that of *Fusarium* which uses the pheromone receptors to detect the presence of potential plant hosts.

Unlike the Ascomycota presented here, all of the Basidiomycota discussed reproduce sexually, whether they are saprophytic or pathogenic. Interestingly, as seen with the Ascomycota, human pathogens like *Cr. neoformans* sexually reproduce through outcrossing most infrequently, although the importance of meiosis for this organism is clear given the ability to self-fertilize. Also, as is the case for the sac fungi, basidiomycete fungi mate in the lab when exposed to potential sources of DNA damage, such as nutrient deprivation. Although these species engage in sexual reproduction, the extent to which outcrossing occurs is more inconsistent within the group. *U. maydis*, a model organism for the group, must find a compatible mating partner in order to enter its host plant. Such is also the case for *U. hordei* and *Sp. reilianum.* The two latter fungal pathogens have each evolved individual strategies for increasing the probability of successful mating, such as chromosomal rearrangements returning to a bipolar mating system (*U. hordei*) and increased mating types (*Sp. reilianum*). In any of the three cases, mating is essential for the organism to enter a host and complete its respective lifecycle.

For other organisms, outcrossing has become less essential although mating remains an integral step in the completion of the lifecycle. For example, *Mi. lychnidis-dioicae* must mate to infiltrate its host plant but mating occurs between members of the same meiotic tetrad. Like the ascomycete fungus *Ca. albicans*, these organisms retain all of the benefits of genomic repair through sexual reproduction and none of the drawbacks of outcrossing. At the other extreme, the mushroom species of the Basidiomycota have evolved a multitude of allelic variations at all components of their mating type loci, resulting in thousands of different “genders” or potential mating partners. In this way, they increase the probability of any two haploid cells being compatible for mating and increase the diversity of the species. It is of note that these organisms are generally saprophytic and are not subject to the selective pressures of co-evolving within a plant or mammalian host.

While mating type loci are present in all members of the dikaryotic fungi, the extent to which they play a role in the individual organism’s lifecycle seems to depend largely on whether or not the fungus interacts with a host. In the Ascomycota, saprophytic organisms rarely mate, while in the Basidiomycota, saprophytic organisms mate more than pathogenic organisms and create extensive species diversity through differences in the mating type loci. Pathogenic ascomycete fungi rarely mate because of the possibility of evoking immune response from the host, while most plant pathogenic basidiomycete fungi must mate in order to access their hosts. Because the mating type loci are maintained in all of these dikaryotic fungi, their retention undoubtedly provides a fitness advantage over those organisms who have lost this genetic information. The most plausible reason for maintaining the ability to mate seems to be repairing DNA damage caused by a variety of conditions, through recombination that occurs during meiosis. In the evolutionary arms race, for mammalian pathogenic species, the use of the mating type loci may have waned in favor of avoiding immune response. However, throughout the course of co-evolution, while the organisms reproduce sexually at a much lower frequency than other members of the dikaryotic fungi, maintenance of the mating type loci has been favored by selection or it would have disappeared in the organisms that rarely use it. Of course, there are exceptions such as *P. carinii*, which is likely to have a sexual component of its lifecycle and *Ca. albicans*, which may reproduce sexually in certain areas of the host body. For plant pathogens, the mating type loci for ascomycete fungi have basically become vestigial genetic information in some species, either not used or repurposed; in contrast, for basidiomycete fungi, sexual reproduction is an essential element of plant pathogen host penetration. However, in all cases, the maintenance of mating type loci in dikaryotic fungi is associated with their fitness relative to organisms that lose this genetic information.

Potentially the strongest evidence for the evolution of meiosis as a means of DNA damage repair in fungi is the existence of homothallic or “self” mating systems. If the selective advantage of maintaining a meiotic pathway were purely for creating variation, this strategy allowing for meiosis without increasing variation would not be maintained with this group of fungi. Rather, it seems that increase in variation was a fortunate byproduct of a system originally evolved to handle stressful environmental conditions. Not all members of either group have been studied extensively and much remains to be learned about the function of mating type loci in the remaining dikaryotic fungi. The organisms included in this review are by no means a complete list of all the interesting and sometimes elusive strategies of sexual reproduction within dikaryotic fungi. Analysis of these organisms does provide insight into the complexity of sexual reproduction in all eukaryotes, especially as it is related to ecological niche. Nearly all organisms reviewed contain at least part of the mating type loci, even those with the most frugal genomes, indicating the ancestral state as well as the importance of mating in the evolutionary history of these organisms. Strong evidence suggests that mating and nutrient deprivation are closely linked, potentially as a way to survive adverse environmental conditions. Examples of increased variation as well as virtually clonal populations indicate that mating and sexual reproduction have not always been used for the same purpose within this group of fungi. Thus, much remains to be learned about the function of sex in eukaryotes as a whole.

## Ethics Statement

The authors declare that this manuscript follows the Ethical Responsibilities of Authors, as indicated in the Instructions to Authors (including not being previously published or simultaneously submitted elsewhere, and consent of all authors).

## Author Contributions

Both authors listed have made a substantial, direct and intellectual contribution to the work, and approved it for publication. RW and MP developed the concept of the review. RW did the bulk of the initial literature research, figure production, and writing. MP helped with overall organization and editing to produce the final product.

## Conflict of Interest Statement

The authors declare that the research was conducted in the absence of any commercial or financial relationships that could be construed as a potential conflict of interest.
